# Physical Activity, Positive and Negative Symptoms of Psychosis, and General Psychopathology among People with Psychotic Disorders: A Meta-Analysis

**DOI:** 10.3390/jcm11102719

**Published:** 2022-05-11

**Authors:** Ernest Swora, Monika Boberska, Ewa Kulis, Nina Knoll, Jan Keller, Aleksandra Luszczynska

**Affiliations:** 1 Wroclaw Faculty of Psychology, SWPS University of Social Sciences and Humanities, 53238 Wroclaw, Poland; mboberska@swps.edu.pl (M.B.); ekulis@swps.edu.pl (E.K.); 2 Department of Education and Psychology, Freie Universität Berlin, 14195 Berlin, Germany; nina.knoll@fu-berlin.de (N.K.); jan.keller@fu-berlin.de (J.K.); 3 The Melbourne Centre for Behavior Change, School of Psychological Sciences, University of Melbourne, Melbourne, VIC 3010, Australia

**Keywords:** physical activity, intervention, psychotic disorder, schizophrenia, meta-analysis, general psychopathology

## Abstract

Objective: Existing reviews provided evidence for the associations between higher physical activity (PA) and lower negative symptoms of psychosis among people with schizophrenia. This meta-analysis goes beyond existing syntheses and investigates associations between PA, positive and negative symptoms of psychosis, as well as symptoms of general psychopathology (referring mostly to cognitive functioning) among people with schizophrenia, but also other psychotic disorders. The moderating roles of the type of diagnosis and the type of exercise intervention were explored. Methods: The study was registered with PROSPERO (CRD42018118236). Six electronic databases were searched; *n* = 27 experimental and observational studies were included, and psychotic symptoms-related data were recorded in one direction (higher values indicate better mental health and lower symptomatology). Results: Higher levels of PA (or participating in PA interventions) were associated with better mental health, that is, lower levels of positive symptoms (all studies: *r* = 0.170; experimental studies: *SMD* = 0.677), negative symptoms (all studies: *r* = 0.214; experimental studies: *SMD* = 0.838), and general psychopathology (all studies: *r* = 0.451; experimental studies: *SMD* = 1.511). The type of diagnosis (schizophrenia vs. other psychotic disorders) did not moderate these associations. Conclusions: We found a consistent pattern of associations between higher levels of PA and lower positive, negative, and general psychopathology symptoms in people with schizophrenia and those with other psychotic disorders.

## 1. Introduction

In the general population, the median lifetime prevalence of psychotic disorders is approximately 0.8% (for a meta-analysis see [1]). Psychosis is manifested with positive symptoms (PS), such as delusions or hallucinations, and negative symptoms (NS), such as apathy, isolation, or lower social functioning [2]. Adjunct psychopathology includes poor attention, a lack of judgement and insight, poor impulse control, or disorientation [3]. Psychotic disorders are associated with several poor clinical outcomes, such as elevated utilization of health and social services and a high risk of disability, as well as negative physical health outcomes, including heart disease, back/neck problems, or headaches [4]. Compared to the general population, people with psychotic disorders are also at risk for low levels of physical activity (PA) and more time spent in sedentary behavior [5]. In people with psychotic disorders, reduced PA levels are among the key predictors of weight gain and symptoms of metabolic syndrome, which in turn contribute to higher mortality [6]. PA interventions delivered to people with psychotic disorders account for exercises of various types and various intensity levels, from light intensity exercises involving yoga and tai-chi to moderate-intensity aerobic exercises, and high-intensity anaerobic training [5,6,7,8].

Besides effects on physical health, accumulating evidence points to positive effects of PA interventions on negative and positive symptoms of psychosis and quality of life [7,8]. In line with the biopsychosocial model of health [9], PA operates via biological, social, or psychological mediators and has the potential to affect mental health outcomes. Research conducted in the general population showed that regular PA is associated with improvement in cognitive functioning, reduced negative affect, a delay in age-related cognitive decline, and slower neurodegeneration [10]. 

Among others, these processes are mediated by PA-induced increases in growth factors such as brain-derived neurotropic factor (BDNF), resulting in an increase in neurogenesis in the hippocampus and enhanced synaptic plasticity [10]. Importantly, low levels of BDNF are associated with higher levels of symptoms of schizophrenia [11]. The associations between BDNF and psychosis symptoms may be particularly salient in the area of cognitive functioning (e.g., processing speed, memory, and executive function; for a review see [12]). In addition to physiological links between PA and symptoms of psychosis, psychosocial factors may mediate the BDNF—psychosis symptoms association. PA interventions usually involve behavioral activation and elements of social skills training which are the core elements of psychological interventions for patients with psychotic disorders [13]. Furthermore, in line with the strength model of self-regulation [14], it may be assumed that engaging in regular PA can bolster various self-control reserves and improve self-control performance. In turn, poor self-control is linked with poorer mental health outcomes via impairments in executive functions (e.g., [15]). In sum, research evidence and theories point towards complex links between lower PA and poorer mental health that may be mediated by physiological (e.g., BDNF-related neuroplasticity) and psychological processes (e.g., self-control).

Several systematic reviews already investigated associations between PA and the level of symptoms among people diagnosed with psychosis. A meta-analysis by Dauwan et al. [7] included controlled trials conducted among people with schizophrenia and found that taking part in a PA intervention resulted in significantly lower levels of positive and negative symptoms as well as lower general psychopathology symptoms. This study analyzed the type of PA training and found that yoga may outperform aerobic training in terms of a reduction of general symptoms [7]. Another meta-analysis of controlled trials among people with schizophrenia [16] indicated that aerobic exercises reduced positive and negative symptoms whereas non-aerobic exercises did not reduce positive or negative symptoms. A third meta-analysis focused on people with schizophrenia only and reviewed findings accumulated in controlled trials on the effects of participation in a PA intervention (aerobic exercises vs. mind-body interventions, e.g., yoga) on negative symptoms only [17]. Medium effects were found for mind–body exercise interventions and small effects were observed for aerobic exercise interventions. A fourth meta-analysis [18] found significant, small-to-moderate effects of mind–body interventions (including yoga, tai-chi, qi-gong, but also mindfulness-based treatment) on negative symptoms among people with schizophrenia. The fifth is the only meta-analysis [19] investigating the effects of PA interventions among people with any psychotic disorders (including schizoaffective disorders, first episode psychosis, etc.). Significant effects of structured PA training (supervised, controlled, and energy expenditure raising) reduced the level of symptom severity (a combination of negative, positive, and general symptoms). Unfortunately, the differences between people with schizophrenia and people with other psychotic disorder were not tested [19].

Existing reviews and meta-analyses leave several questions unanswered. First, meta-analyses published to date focused on people with schizophrenia [7,16,17,18], and there is no synthesis of evidence for the associations between PA and symptoms of psychosis among people with other psychotic disorders. Second, associations between PA and positive symptoms were analyzed in only two reviews [7,16], with one indicating significant associations and the other showing that the average effects were not significant. An additional synthesis of associations between positive symptoms of psychosis and PA is thus called for. Third, the associations between PA and adjunct symptoms characteristic of psychotic disorders (henceforth: general psychopathology; [3]), such as poor attention, a lack of judgement and insight, poor impulse control, and disorientation, were tested in the meta-analysis dealing with people with schizophrenia, published in 2016 [7]. An update of these findings [7] and insights into the associations between PA and general psychopathology among people with other psychotic disorders are needed. Two previous meta-analyses compared aerobic exercises with other exercises [7,16] and yielded inconsistent results for various “non-aerobic” PA interventions. Consequently, further clarifications of the effects of different types of PA training are needed. Finally, the focus to date was on intervention studies, whereas observational studies showing longitudinal or cross-sectional associations were not summarized in a systematic manner.

The present study aims at synthesizing empirical evidence for associations between levels of PA among people with psychotic disorders and three types of symptoms of psychosis: positive, negative, and adjunct symptoms of general psychopathology in psychotic disorders. We hypothesized that higher levels of PA would be associated with lower levels of the three types of symptoms. We also tested if participation in PA interventions would result in lower levels of the three types of symptoms, compared to changes observed in control conditions without any PA training. The role of potential moderators was explored across the studies varying in (1) the type of the diagnosis (schizophrenia vs. individuals with other types of psychotic disorders); (2) the type of PA interventions (aerobic vs. other types of PA interventions). We explored the moderating effects of the methodology of original studies, such as the design of the study (experimental with follow-ups vs. observational with an intervention but without a control group, longitudinal without an intervention, versus observational-cross-sectional), the quality of the study (high, moderate, or low), and the type of measurement of psychotic symptoms (PANSS vs. other assessment instruments).

## 2. Method

The study was conducted in accordance with PRISMA guidelines [20] and registered with the PROSPERO database (International Prospective Registered Systematic Reviews database), reference number CRD42018118236. In total, six electronic databases were searched: PsycINFO, PsycARTICLES, Academic Search Complete, Health Source: Nursing/Academic Edition, Scopus, and MEDLINE. The searched terms included: PA-related terms (e.g., ‘physical activity’ OR ‘exercise*’ OR ‘sport’), intervention-related terms (e.g., ‘intervention’ OR ‘program’ OR ‘treatment’), and psychosis-related terms (e.g., ‘psychosis’ OR ‘schizophrenia’ OR ‘psychotic disorder*’). These terms were chosen based on keywords used in reviews addressing PA and psychotic symptoms [7,17,18].

**Figure 1:**
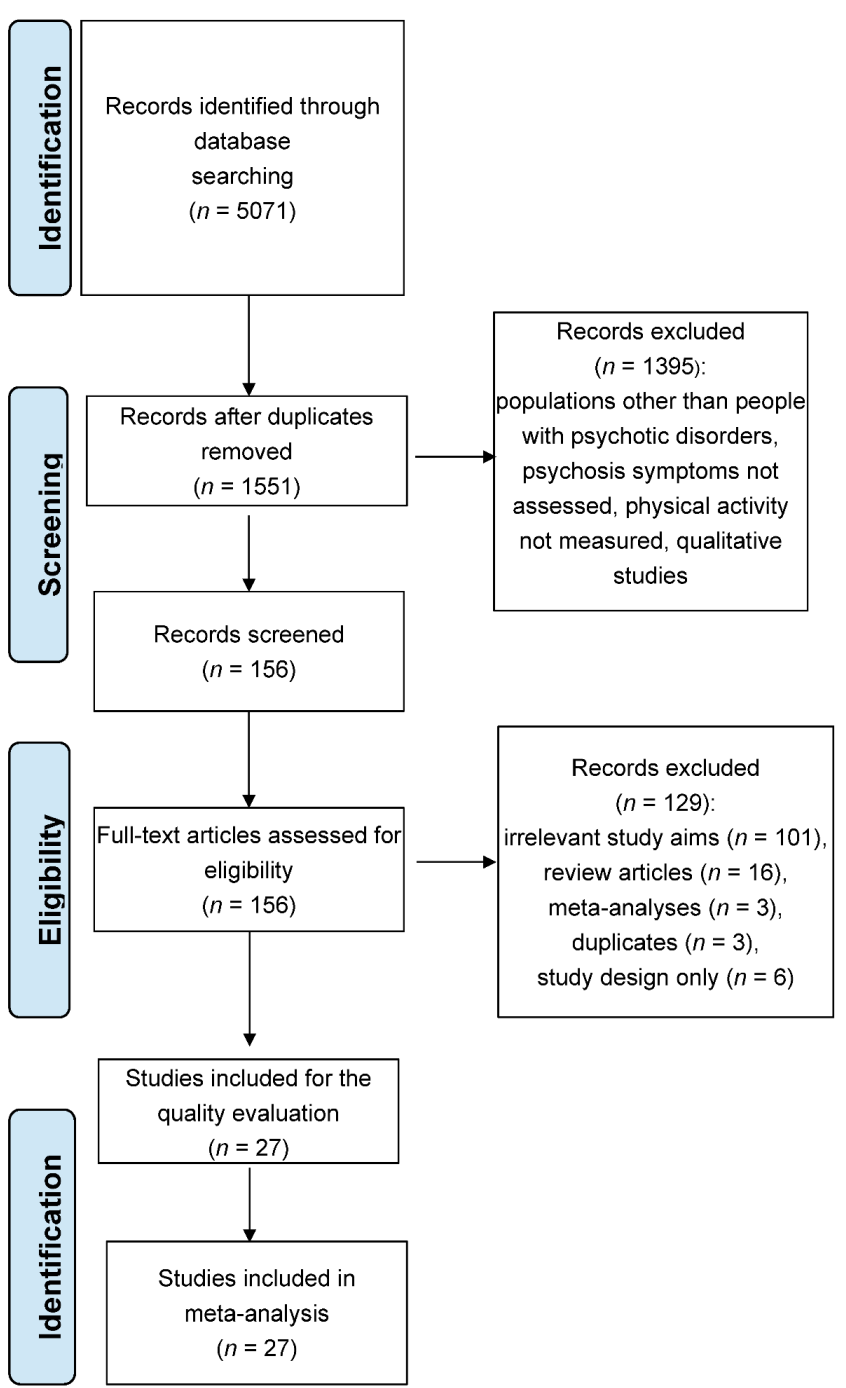
The flow chart of the selection process.

The study flow is presented in Figure 1. Overall, *k* = 27 original studies were included in the meta-analysis; 23 of them reported associations between PA and positive and negative symptoms, 3 studies reported associations between PA and negative symptoms only, and 1 study reported an association between PA and general psychopathology only. A total of six studies included 2 independent samples enrolled in different PA interventions [6,21,22,23,24]. A total of 33 samples obtained from 27 studies were included: 31 samples (from 26 studies) testing associations between PA and negative symptoms; 28 samples (from 23 studies) addressing associations between PA and positive symptoms; 19 samples (from 17 studies) testing PA—general psychopathology associations.

In case the results from one and the same study were presented in two papers, the study with a larger sample or the more recent publication (in case the sample sizes were the same) was included.

Across the stages of data search, data extraction, quality evaluation, and data coding, three independent researchers (ES, MB, and EK) were involved. During the data search, three researchers read abstracts, keywords, and titles in order to establish if the paper reported an original study accounting for the associations between PA and psychotic symptoms, extracted the respective data independently, coded the data, and evaluated the quality of the original study. Any discrepancies during the process of data extraction and quality evaluation were resolved by a consensus method (Higgins et al., 2019), involving a discussion between three researchers (ES, MB, and EK) and the fourth researcher (AL). In case of a discrepancy between the researchers (ES, MB, and EK), the fourth researcher (AL) retrieved respective data independently, conducted the quality evaluation and led the discussion aiming at a consensus.

### 2.1. Inclusion/Exclusion Criteria

The main inclusion criteria were: (1) original studies reporting associations between PA and psychotic symptoms; (2) PA was assessed quantitatively with either self-report instruments or an objective measure (e.g., accelerometer); (3) psychotic symptoms were assessed quantitatively, without restrictions referring to the type of psychotic disorder or its stage (i.e., acute vs. chronic) or duration of positive or negative symptoms; (4) participants were diagnosed with psychotic disorders (schizophrenia, schizoaffective disorders, bipolar disorders, or first-episode psychosis); (5) participants were at least 18 years old; (6) studies published in English in peer-reviewed journals.

The following exclusion criteria were applied: (1) studies including other populations than patients with a diagnosed psychotic disorder or first-episode psychosis; (2) studies that did not measure psychotic symptoms but included an assessment of anxiety, psychological distress, or quality of life; (3) experimental research with no adequate information about the PA intervention (e.g., indicating only the presence of a PA component in the treatment, without any further specification about the actual content and delivery of the PA intervention); (4) correlational research providing no assessment of PA. 

### 2.2. Data Extraction and Quality Assessment

Extracted data (see Table 1) included details of PA and psychotic symptoms measurement, sample characteristics, and the main findings of the original study. Statistical information and data necessary to conduct the quality evaluation were also retrieved. When the data required to conduct a meta-analysis were not included in the original paper, the research team attempted to contact the authors via e-mail (e-mails sent up to 3 times) and requested the required data.

The Quality Assessment Tool for Quantitative Studies [25] was applied to evaluate the quality of identified studies. This tool addresses such criteria as selection bias, study design, confounders, blinding, data collection methods, withdrawals/drop-outs, or intervention integrity. Each criterion was rated using a 3-point response scale (1 = ‘strong’, 2 = ‘moderate’, 3 = ‘weak’), with the overall rating varying from 1 (high quality) to 3 (low quality).

### 2.3. Coding

For the purpose of this review PA was defined as any movement characterized by an energy expenditure above 1.5 metabolic equivalents. PA was coded as objectively measured if it was assessed with accelerometers, pedometers, or position activity electronic loggers. Self-report measurements of PA included questionnaires, such as International Physical Activity Questionnaire (IPAQ; [49]) or interview methods (Table 1). PA interventions were defined as treatments targeting PA promotion of MET ≥ 1.5 (with bouts > 10 min).

Psychotic symptoms were coded into three types: positive, negative, and general psychopathology in psychotic disorder. Positive symptoms were defined as delusions, hallucinations, or bizarre behavior, while negative symptoms included symptoms such as autistic thinking, withdrawal, passivity, and isolation [2]. The applied measures of positive and negative symptoms included questionnaires such as the Positive and Negative Symptoms Scale (PANSS) [3] or structured interviews (see Table 1). General psychopathology in psychotic disorders was coded as a construct specifically measured with PANSS [3]. General psychopathology addresses the severity of the disorder and its adjunct characteristics, including poor cognitive functioning (e.g., poor attention), a lack of judgement and insight, poor impulse control, and motor retardation.

In terms of the diagnosis, the study was coded as dealing with patients with a psychotic disorder if the reported findings were obtained in samples consisting solely of people diagnosed with a psychotic disorder who were treated with pharmacotherapy and or/psychotherapy. Studies conducted in the general population with a purpose of screening for psychotic symptoms were excluded. There were no studies that examined samples combining a general population and people with a diagnosis of a psychotic disorder. Finally, the studies were coded as enrolling people diagnosed with schizophrenia or diagnosed with other psychotic disorders (including schizoaffective disorders and/or other types of psychosis).

The type of the PA intervention was coded as belonging to one out of five categories: aerobic exercises (if the focus of the intervention was to conduct aerobic exercises); yoga (if the intervention included yoga training only); high-intensity interval training (HIIT, involving repeated bouts of high-intensity exercise intersected with recovery periods); psychoeducation (if the focus was mostly on a psychosocial intervention, including PA guidelines and examples of exercises); ‘other exercise interventions’ (if various types of exercise were combined in one training, without specifying the type of training or its intensity/duration).

Regarding the design, studies were coded as ‘experimental-longitudinal’ if patients were assigned to a PA intervention versus a control group (without a PA-related treatment) and reported longitudinal data. The ‘experimental-longitudinal’ studies included randomized controlled trials and controlled trials. Studies were coded as ‘observational-longitudinal’ if they reported longitudinal associations between PA and psychotic symptoms, either without any intervention, or using a pre-post measurement with a PA intervention (i.e., all participants assigned to a PA intervention). Studies were coded as ‘observational—cross-sectional’ if they reported cross-sectional associations between PA and psychotic symptoms (even if the original study reported a controlled trial addressing other outcomes than PA, but PA and psychotic symptoms were reported only at baseline, the study was coded as ‘observational—cross-sectional’).

### 2.4. Data Synthesis and Analysis

In order to calculate the estimates of the average effects, heterogeneity, and the effects of the moderators, data obtained from 27 original studies were meta-analyzed using the Comprehensive Meta-Analysis software, version 2.2 [50]. The meta-analysis accounted for original research providing bivariate association coefficients, obtained in an equation without covariates. In the case of experimental-longitudinal studies, means and standard deviations for baseline and post-intervention PA levels were used to calculate standardized mean difference (SMD) coefficients. Pearson correlations were used as the effect size indicator for analyses conducted for all included studies. Correlations were synthesized to form the cumulative effect size by transformation into Fisher’s *z* (see [50]). In the case respective coefficients were not provided, authors were contacted by e-mail. In total, ten correlation coefficients were obtained from the authors’ e-mail replies. In case of studies comparing groups with different levels of PA, data regarding levels of psychotic symptoms in each group were obtained and compared. In the case of observational-longitudinal studies, coefficients representing the associations between baseline and the latest available follow-up were included into the analysis. For all studies, the original coefficients were coded in one direction, with positive values indicating that higher PA levels were associated with better mental health (i.e., less negative or positive symptoms, lower general psychopathology). 

An overall effect was determined based on coefficients available in all original studies included in the meta-analysis (*k* = 27), using a respective indicator of positive symptoms (*k* = 28 samples from 23 studies), negative symptoms (*k* = 31 samples from 26 studies), or general psychopathology (*k* = 19 samples from 17 studies). Next, the estimates of average effects were calculated for data obtained in controlled trials only, using an indicator of positive symptoms (*k* = 12 samples from 9 studies), negative symptoms (*k* = 12 samples from 9 studies), and general psychopathology (*k* = 8 samples from 7 studies). We performed moderation analyses to investigate differences in estimated effects depending on the type of diagnosis (schizophrenia vs. other psychotic disorders); the type of a PA intervention, the type of measurement of psychotic symptoms (PANSS vs. other); the study design (experimental-longitudinal vs. observational-longitudinal vs. observational—cross-sectional); and the quality of studies included. The moderation analyses were conducted if the number of respective subgroups was *k*  ≥  2. The estimate of the effect size was calculated for each level of a moderator. Group mean effect sizes were compared using the *Qʙ* statistic, which is an omnibus test for detecting between-group differences [50]. 

A random-effects model was used to calculate the estimates of the effect size. To investigate an asymmetry that may be caused by the publication bias, the respective funnel plots were screened (see Supplementary material 1, Figures S1–S6) and Egger’s tests were conducted. To assess heterogeneity, *Tau* and *I*^2^ values were calculated [50]. Data affiliated with this study are available at Open Science Framework (see https://osf.io/42cdz/, Accesses on 9 May 2022).

## 3. Results

### 3.1. Characteristics of Included Studies and Risk of Bias Evaluation

This section may be divided into subheadings. It should provide a concise and precise description of the experimental results, their interpretation, as well as the experimental conclusions that can be drawn.

Descriptive information about included studies is summarized in Table 1. A total of 1664 participants were enrolled in *k* = 27 studies, with sample sizes ranging from 8 to 187 people (*M* = 51.26, *SD* = 41.6) and ages ranging from 24 to 65 years old. In total, twenty-one studies (77.7%) enrolled people diagnosed with schizophrenia only, and the remaining six studies (22.3%) enrolled patients diagnosed with schizoaffective disorder, bipolar disorder, or first-episode psychosis. Across studies, all participants were receiving pharmacotherapy. 

Almost half of studies applied an observational-longitudinal design (*k* = 13, 48.15%), 9 (33.3%) were experimental-longitudinal, and 5 (18.5%) were observational—cross-sectional. Regarding positive and negative symptoms, the majority of studies (*k* = 22, 81.5%) relied on the PANSS scale [3], whereas 5 (18.5%) used other symptom assessment tools. All 17 studies which addressed general psychopathology used the PANSS [3]. In 20 studies (74%), a PA intervention was conducted (including both experimental and pre-post observational studies). Only 5 studies used an ‘objective’ assessment of PA or physical fitness (accelerometer: *n* =  2, 7.4%; 6 min walk test: *n* = 3, 11.1%); and the remaining studies applied self-reports to measure PA. 

Original studies were conducted in 17 different countries. Most frequently, the studies were conducted in the United States of America (*n* = 3, 11.1%) and Taiwan (*n* = 3, 11.1%). Two studies (7.4% each) were conducted in each of the following countries: Belgium, China, India, Japan, the Netherlands, United Kingdom. Only one study came from Australia, Brazil, Czech Republic, Canada, Greece, Iran, Portugal, South Korea, and Turkey, respectively.

Regarding quality evaluation, 25.9% (*n* = 7) of the included studies have been rated as representing a high level of quality [25], 16 (59.2%) studies were rated as of moderate level of quality, and 4 (14.8%) represented a low level of quality. The risk of bias analysis indicated no tendency to obtain greater effects in small studies, for negative symptoms (Egger’s test: *hc* = 0.99; *p* = 0.129) and general psychotic psychopathology (Egger’s test: *hc* = 0.84; *p* = 0.315). In the case of positive symptoms, findings indicated that larger effects were obtained in smaller samples (Egger’s test: *hc* = 1.68; *p* = 0.026). See Supplement 1 for funnel plots.

### 3.2. Associations between PA and Positive, Negative, and General Psychopathology Symptoms

The estimate of the overall average effect for the association between indicators of PA and *positive symptoms* (*k* = 28 samples), including experimental-longitudinal, observational-longitudinal, and observational—cross-sectional studies, was significant and small, with a weighted *r*  = 0 .17, 95% CI [0.02; 0.31], *p*  <  0.027, suggesting that higher levels of PA were associated with more beneficial profiles of positive symptoms (i.e., a lower levels of positive symptoms; Table 2). To demonstrate how much an effect might vary across different populations, prediction intervals were calculated with *τ*  =  0.33 [50]. It can be expected that in 95% of the different populations, the true correlation will fall in the approximate range from −0.51 to 0.82. The analysis as repeated for the experimental--longitudinal studies (*k* = 12 samples) showed a significant average effect of PA on positive symptoms, *SMD*  =  0.68, 95% CI [0.22; 1.13], *p*  <  0.004, indicating that participants of PA interventions showed better positive symptom profiles (i.e., had less positive symptoms at follow-ups, compared to patients assigned to the control group (Table 3).

The estimate of the overall average effect for the association between indicators of PA and *negative symptoms* (*k* = 31 samples), including experimental-longitudinal, observational-longitudinal, and observational-cross-sectional studies, was significant and small, with a weighted *r*  =  0.21, 95% CI [0.08; 0.34], *p*  =  0.002, suggesting that higher levels of PA were associated with more beneficial profiles of negative symptoms (e.g., levels of negative symptoms; Table 2). Based on *τ* of 0.32 [50], the calculation of prediction intervals suggests that it may be expected that in 95% of the different populations the true correlation will fall in the range from −0.45 to 0.78. The significant average effect of PA on *negative symptoms* was also found in the analysis of the 12 experimental-longitudinal studies, weighted *SMD*  =  0.84, 95% CI [0.23; 1.45], *p*  <  0.007. Participants of PA interventions showed better negative symptom profiles (i.e., had fewer negative symptoms at follow-ups), compared to participants from the control groups (Table 3). 

The average effect for the association between indicators of PA and *general psychopathology* index in psychosis (*k* = 19 samples), including experimental-longitudinal, observational-longitudinal, and observational-cross-sectional studies was significant and moderate with a weighted *r*  = 0.45, 95% CI [0.21; 0.64], *p*  < 0 .001, suggesting that higher levels of PA were associated with better functioning in the area of general psychopathology (i.e., less general psychopathology symptoms; Table 2). Based on a *τ*  = 0.54 [50], the calculation of prediction intervals suggests that it may be expected that in 95% of the different populations the true correlation will fall in the range from −0.46 to 0.89. The significant average effect of PA on general psychopathology was also found in the analysis of the experimental–longitudinal studies only (*k* = 8 samples), with a weighted *SMD* = 1.51, 95% CI [0.68; 2.34], *p*  < 0.001. Participants of a PA intervention had better functioning in the area of general psychopathology follow-ups, compared to patients assigned to the control groups (Table 3).

### 3.3. Moderating Effects: The Diagnosis, the Type of PA Intervention, Studies’ Design and Quality

Moderation analyses were performed to address potential sources of heterogeneity for the three indices of *psychotic symptoms* (Table 2). The first set of moderation analyses was conducted for associations between PA and *positive symptoms* (Table 2). There was no significant moderating effect of the type of diagnosis in studies enrolling people with schizophrenia vs. those enrolling people with other psychotic disorders (*p* = 0.845). The types of PA interventions (exercises, aerobic exercises, yoga, and high-intensity interval training) showed no significant moderating effect (*p* = 0.700). Additionally, the comparisons conducted between any two types of exercise interventions yielded no significant differences (all *ps* > 0.245). Another analysis comparing yoga interventions vs. exercise, aerobic, or HIIT interventions yielded no significant difference (*p* = 0.280). There was no significant moderating effect on the type of measurement (PANSS vs. other scales, *p* = 0.063). The study design had significant moderating effects (*p* = 0.001), however, with stronger effects found for observational-longitudinal or experimental-longitudinal studies, compared to observational--cross-sectional studies. The quality of included studies did not moderate the effect size (*p* = 0.271).

The second set of moderation analyses was conducted for associations between PA and *negative symptoms* (Table 2). There was no moderating effect on the type of diagnosis (*p* = 0.640). The type of PA intervention did not moderate the effect (*p* = 0.121), but exploratory two-group comparisons indicated significantly larger effects of aerobic training compared to psychoeducation (*p* = 0.018) and larger effects of HIIT compared to psychoeducation (*p* = 0.043). There were no significant moderating effects of the type of measurement of psychotic symptoms (*p* = 0.624). There was, however, a significant moderating effect of the study design (*p* = 0.003). The comparisons conducted for two types of design indicated that significantly stronger associations were found for observational –longitudinal studies compared with observational–cross-sectional studies (*p* = 0.001) and for experimental-longitudinal studies compared to observational–cross-sectional studies (*p* = 0.035). There was also a significant difference between the studies in terms of study quality as a moderator (*p* = 0.001). Exploratory two-group comparisons indicated that studies of weak quality yielded stronger effects than studies of moderate quality (*p* < 0.001). The final set of analyses investigated the effects of the moderators on the average effects obtained for the association between PA and the *general psychopathology* index (Table 2). There was no moderating effect of the type of diagnosis (*p* = 0.607). The comparisons between the four types of PA interventions showed significant moderating effects (*p* = 0.008). Exploratory two-group comparisons indicated significantly stronger effects of the yoga interventions compared to exercise interventions (*p* = 0.001) and yoga interventions compared to HIIT interventions (*p* = 0.003). However, these significant effects were obtained in comparisons conducted in a small number of studies, enrolling small samples (2 to 4 studies per a comparison arm, samples varying from 45 to 95 individuals; Table 1). Compared to exercise, aerobic, or HIIT interventions combined, yoga or psychoeducation interventions yielded similar effects (*p* = 0.905). There were no moderating effects of the study design (*p* = 0.590) or the quality of the study (*p* = 0.326).

## 4. Discussion

This study provides an overarching synthesis of associations between PA and symptoms of psychosis in people with psychotic disorders. In addition to negative and positive symptoms, our study systematically tested the associations of PA with general psychopathology (poor cognitive functioning, a lack of judgement and insight, poor impulse control, and disorientation [3]). In particular, we found a significant moderate association between higher PA and better mental health in the domain of general psychopathology. Compared to previous meta-analyses focusing solely on people with schizophrenia, we tested respective associations also among people with other psychotic disorders. The associations between PA and positive, negative, and general psychopathology symptoms were similar, regardless of the type of diagnosis.

Regarding *positive symptoms*, to date, two reviews addressed their relationships with PA among people with schizophrenia [7,18], and their findings were partly contradictory. Our study shows that the associations between PA and positive symptoms are weak but significant, and, additionally, that the associations found among people with other psychotic disorders are similar to those obtained in research enrolling people with schizophrenia. Our findings also indicate that the type of applied measurement may be a source of significant differences in obtained associations. In particular, we detected that larger effects may be obtained with the PANSS, compared to other measures of psychotic symptoms. The PANSS is relatively long and may be difficult to complete for patients with more severe symptoms [51]. Thus, it is possible that research using the PANSS may be conducted mostly among people with mild to moderate symptoms. It may thus be assumed that PA’s beneficial effects on positive symptoms may solely be true for people diagnosed with a psychotic disorder of mild to moderate severity. Regarding the relationship between PA and *negative symptoms* among people with a psychotic disorder, we found that the estimates of average effects were significant but small. The values of the obtained effects are in line with results from previous reviews, addressing experimental studies conducted among people with schizophrenia [7,16,17,18]. We also found that the design of the study and its quality may moderate the effect sizes observed for negative symptoms. These effects should, however, be placed in a broader methodological context. For example, observational—cross-sectional studies (with the smallest effects) were enrolling the largest samples, which could affect the size of the weighted *r* coefficient. Lower quality in studies yielding larger effects than those of higher quality confirms that the low quality of the original studies may result in biased (exaggerated) findings [25]. In sum, larger effects were more likely to be observed in poorer quality studies, which were also likely to enroll small samples. The observed effects might result from a bias related to low quality and small sample sizes, and consequently, low precision of these particular meta-analytic findings [52]. 

To date, only one meta-analysis [7] showed significant associations between PA and *general psychopathology* symptoms (e.g., poor attention, a lack of judgement and insight, poor impulse control, and disorientation; see [3]) among people with schizophrenia. We found that among people with any psychotic disorders higher PA was moderately associated with better mental health (lower levels of general psychopathology symptoms). Based on our findings, it may be assumed that the associations between PA and general psychopathology (weighted *r* = 0.45) may be larger than those observed for PA and positive/negative symptoms (weighted *r*s of 0.17 to 0.21). This is in line with research linking cognitive functioning in psychosis and BDNF, the neurotropic factor affected by PA. Low BDNF is typical among people with psychotic diseases [12]. BDNF levels may be increased through regular PA [10]. Recent research emphasizes that the associations between BDNF levels and schizophrenia symptoms, but mostly regarding symptoms, are related to poor cognitive functioning (e.g., working memory, attention, processing speed, motor function, and executive function; for review see [12]). Symptoms related to poor cognitive functioning are best captured by the general psychopathology symptoms of psychosis. 

The findings indicating significant associations between PA and all three types of symptoms investigated across the samples of patients with either schizophrenia or other psychotic disorders might suggest that PA has an unspecific, generalized effect on the functioning of people with psychotic disorders. The unspecific effects of PA on various symptoms of psychosis might result from interactions between PA and changes in brain function and structures, such as the sensorimotor system. Sensorimotor system abnormalities are related to a variety of positive and negative symptoms, and abnormalities in psychomotor functions [53,54]. Early sensorimotor abnormalities are vulnerability markers and predictors of the poor clinical course in patients with first-episode psychosis [53]. Furthermore, alterations in cross-network connectivity in sensorimotor regions of the brain have been found to be associated with various psychosis-like symptoms, including altered intrinsic and extrinsic self-processing (agency and identity), auditory hallucinations, disrupted self-recognition, and negative affect [54]. Further research is needed to test if PA interventions may affect the sensorimotor system function, that in turn may result in a general reduction of various symptoms among people with psychotic disorders. 

This study compared people with schizophrenia and people with other psychotic disorders. This distinction is arbitrary, and the optimal solution would be to investigate if the tested associations are similar or distinct when comparing homogeneous groups, meeting the diagnostic criteria of one disorder (e.g., schizophrenia vs. schizoaffective disorder). Such comparisons were not possible due to the limited number of original research conducted among people with a diagnosis other than schizophrenia. The decision to compare the effects of PA among people with schizophrenia vs. other psychotic disorders is rooted in previous research that indicated systematic differences between the two populations. For example, a crucial difference between schizophrenia and all other psychotic disorders, including the closely related schizophreniform and schizoaffective disorders, is a decrease in the level of functioning below the level achieved prior to the onset of psychosis (criterion B for schizophrenia) [55]. Other research shows systematic differences between schizophrenia vs. other psychotic disorders in metabolomics (e.g., levels of carbohydrates, amino acids, organic acids, lipids) with the poorest metabolomics in schizophrenia [56], or in a decline across domains of quality of life, compared to healthy individuals (a decrease in 11 out of 15 domains among patients with schizophrenia; any other psychotic disorders—a significant decrease was found in 3–10 domains) [57]. Metabolomics and quality of life are, in turn, closely associated with PA. 

Our study attempted to explore the moderating effects of the type of PA intervention. Two previous reviews enrolling people with schizophrenia (evaluating positive and negative symptoms) yielded inconsistent findings in this area [16,17]. Overall, we found no effects of the type of the PA intervention on the associations between PA and positive symptoms. In the case of negative symptoms, psychoeducation with examples of PA (but no actual PA training) yielded smaller effects than HIIT or aerobic training. This may be due to a limited involvement in actual PA during the psychoeducation intervention. In the case of general psychopathology, some differences between the types of training (yoga vs. aerobic, HIIT, or other exercise interventions) were observed, suggesting that yoga interventions may result in larger effects. This conclusion is likely to be premature as there were only two studies involving a total of 45 participants; therefore, the effects may be due to a small number of small sample sizes. We encountered difficulty in coding the types of PA interventions, resulting from a lack of protocols describing the type of exercise, their intensity, length and number of bouts, etc. These difficulties resulted in assigning the majority of training to a broad category of ‘other exercise interventions’. Future research should provide detailed intervention protocols, allowing the identification of intervention components that are specific for successful interventions.

This study has several limitations. The majority of the included samples were small, which may result in an increased likelihood of biased findings. The included studies were heterogeneous in terms of populations included (types of treatment applied, age and gender differences, differences in the time since the first diagnosis, etc.) and the types of PA interventions (e.g., from training including mostly high-intensity exercise to low-intensity training); therefore, any conclusions should be treated with caution. Research on psychotic disorders other than schizophrenia is rare; thus, the present findings are preliminary. Across the included studies only one tested the moderating effects of gender, two consisted of men only, one did not provide the distribution of gender, and the remaining 23 studies reported the proportion of men and women but did not test the effects of gender. A lack of testing for potential gender effects in original studies did not allow us for a systematic evaluation if men and women differ in the strength of associations between PA and symptoms of psychosis. Across all included studies, pharmacotherapy was provided to all participants. Unfortunately, original research did not test the role of the type of treatment as a potential moderator of the PA-symptoms associations. Future research should clarify if the links between PA and symptoms of psychosis may vary depending on antipsychotic medication.

## 5. Conclusions

Regardless of its limitations, this study provides a novel synthesis of accumulating evidence. PA and PA interventions are associated with more beneficial profiles of positive and negative symptoms (small effect sizes), but also with better functioning in terms of general psychopathology symptoms, predominantly referring to cognitive functioning (moderate effect sizes). The associations are similar in size among people with schizophrenia and among people with other psychotic disorders. Practitioners, policy-makers, and other stakeholders developing optimal care guidelines should consider PA interventions as a standard component of treatment for patients with schizophrenia and other psychotic disorders.

## Figures and Tables

**Table 1:** Characteristics of the studies included in the meta-analysis.

	First Author, Publication Year	*N* (% Female)	Age Range, Mean (*SD*)	Population	Country	Study Design	PS Measure	Intervention	Results *	Quality Score
1	Acil (2008) [26]	30 (40%)	21–45	Schizophrenia	Turkey	Observational-longitudinal	SANS, SAPS	Aerobic Exercise	Positive: *r* = 0.33; Negative: *r* = 0.35	3
2	Behere (2011) [21]	66 (29%)	EXP: 31.3 (9.3); CT: 30.2 (8.0)	Schizophrenia	India	Experimental-longitudinal	PANSS	Exercise Yoga	EX: Positive: *r* = 0.09; EX: Negative: *r* = 0.15; YA Positive: *r* = 0.16; YA: Negative: *r* = 0.03	2
3	Campos (2015) [27]	32 (25%)	EXP: 39.77 (9.2), CT: 39.00 (5.60)	Schizophrenia	Portugal	Observational-longitudinal	PANSS	Other	Positive: *r* = −0.05; Negative: *r* = −0.06; GP: *r* = −0.04	2
4	Dodd (2011) [28]	8 (25%)	45.9 (10.1)	Schizophrenia	Australia	Observational-longitudinal	PANSS	Aerobic exercise	Positive: *r* = −0.14; Negative: *r* = 0.16; GP: *r* = 0.17	3
5	Firth (2018) [29]	38 (21%)	25.8 (4.6)	Other psychotic disorders	United Kingdom	Observational-longitudinal	PANSS	Exercise	Positive: *r* = 0.25; Negative: *r* = 0.38; GP: *r* = 0.40	2
6	Fisher (2020) [30]	22 (0%)	24.8 (4.8)	Other psychotic disorders	United Kingdom	Experimental-longitudinal	PANSS	Exercise	Positive: *r* = 0.28; Negative: *r* = −0.04; GP: *r* = 0.24	1
7	Gholipour (2012) [31]	45 (0%)	38 (8)	Schizophrenia	Iran	Observational-longitudinal	SANS	Exercise	Negative: *r* = 0.61	3
8	Ho (2016) [22]	151 (46%)	55.0 (7.4)	Schizophrenia	China	Experimental-longitudinal	PANSS	Exercise Tai-chi	Exercises Positive: *r* = −0.11; Exercises Negative: *r* = −0.13; Tai-chi Positive: *r* = −0.38; Tai-chi Negative: *r* = −0.17	2
9	Kaltsatou (2015) [32]	16 (12%)	59.9 (14.1)	Schizophrenia	Greece	Experimental-longitudinal	PANSS	Exercise	Positive: *r* = 0.20; Negative: *r* = 0.14; GP: *r* = 0.18	2
10	Lee (2013) [33]	187 (60%)	37.9 (8.2)	Other psychotic disorders	China	Observational–cross-sectional	SAPS, SANS		Positive CS: *r* = −0.04; Negative CS: *r* = 0.14	2
11	Leutwyler (2014) [34]	30 (40%)	55-older	Schizophrenia	USA	Observational-cross-sectional	PANSS		Negative: *r* = −0.20;	2
12	Manjunath (2013) [23]	88 (44%)	EXP: 31.7 (8.8), CT: 31.1 (7.8)	Schizophrenia	India	Observational-longitudinal	PANSS	Yoga Exercise	Positive: Yoga: *r* = 0.67; Exercise: *r* = 0.59; Negative: Yoga: *r* = 0.57; Exercise: *r* = 0.56; GP: Yoga: *r* = 0.82; Exercise: *r* = 0.72	2
13	Romain (2019) [35]	66 (38%)	30.73 (7.23)	Other psychotic disorders	Canada	Experimental-longitudinal	PANSS	HIIT	Positive: *r* = 0.60; Negative: *r* = 0.78; GP: *r* = 0.89	1
14	Scheewe, Backx (2013) [36]	31 (25%)	29.2 (7.2)	Schizophrenia	Netherlands	Experimental-longitudinal	PANSS	Exercise	Positive: *r* = 0.25; Negative: *r* = 0.13; GP: *r* = 0.26	1
15	Scheewe, van Haren (2013) [37]	84 (26%)	28.5 (7.3)	Schizophrenia	Netherlands	Observational-longitudinal	PANSS	Exercise	GP: *r* = 0.27	1
16	Shimada (2019) [38]	32 ^a^	20–65	Schizophrenia	Japan	Experimental-longitudinal	PANSS, SANS	Aerobic exercise	Positive: *r* = 0.30; Negative: *r* = 0.48; GP: *r* = 0.34	3
17	Shin (2016) [39]	61 (43%)	23–61; 46.59 (8.40)	Schizophrenia	Korea	Observational-cross-sectional	PANSS		Positive: *r* = −0.51; Negative: *r* = −0.36; GP: *r* = −0.46	2
18	Silva (2015) [24]	34 (0%)	Resistance—32.91 (2.28) Aerobic—35.55 (2.63)	Schizophrenia	Brazil	Experimental-longitudinal	PANSS	Aerobic exercise Resistance exercise	Aerobic-positive: *r* = 0.57; Aerobic-negative: *r* = 0.45; Aerobic GP: *r* = 0.77; Resistance-positive: *r* = 0.82; Resistance-negative: *r* = 0.77; Resistance GP: *r* = 0.88	1
19	Su (2016) [40]	22 (46%)	37.64 (8.23)	Schizophrenia	Taiwan	Observational-longitudinal	PANSS	Aerobic Exercise	Positive: *r* = 0.23; Negative: *r* = 0.37	2
20	Svatkova (2015) [41]	81 (26%)	18–48	Schizophrenia	Czech Republic	Experimental-longitudinal	PANSS	Exercise	Positive: *r* = 0.29; Negative: *r* = 0.20 GP: *r* = 0.28	2
21	Takahashi (2012) [42]	23 (48%)	43.5 (11.8)	Schizophrenia	Japan	Observational-longitudinal	PANSS	Aerobic exercise	Positive: *r* = 0.04; Negatve: *r* = 0.09 GP: *r* = 0.12	2
22	Vancampfort (2017) [43]	19 (26%)	24.4 (5.1)	Other psychotic disorders	Belgium	Observational-cross-sectional	PANSS		Positive moderate: *r* = −0.01; Positive vigorous: *r* = −0.31; Negative moderate: *r* = 0.06; Negative vigorous: *r* = 0.11	1
23	Vancampfort (2012) [44]	52 (40%)	35.74 (10.75)	Schizophrenia	Belgium	Observational-cross-sectional	PECC		Positive: *r* = −0.22; Negative: *r* = −0.45	2
24	Van Citters (2009) [45]	76 (72%)	43.5 (11.4)	Other psychotic disorders	USA	Observational-longitudinal	SANS	Exercise	Negative: *r* = 0.07	2
25	Visceglia & Lewis (2011) [46]	10 (40%)	37.40 (13.75)	Schizophrenia	USA	Observational-longitudinal	PANSS	Yoga	Positive: *r* = 0.56; Negative: *r* = 0.43; GP: *r* = 0.64	2
26	Wang (2018) [47]	62 (52%)	38.3 (8.34)	Schizophrenia	Taiwan	Observational-longitudinal	MC-PANSS	Exercise	Positive: *r* = 0.31; Negative: *r* = 0.41; GP: *r* = 0.47	1
27	Wu (2015) [48]	18 (55%)	38.39 (8.24)	Schizophrenia	Taiwan	Observational-longitudinal	PANSS	HIIT	Positive: *r* = -.01; Negative: *r* = 0.31; GP: *r* = 0.30	2

*N* = sample size; EXP = the experimental group; CT = the control group; PA = physical activity; PS = psychotic symptoms; Positive = positive symptoms of psychosis; Negative = negative symptoms of psychosis; GP = general psychopathology (total score); PANSS = Positive and Negative Syndrome Scale; MC-PANSS = Mandarin Chinese Version of the Positive and Negative Syndrome Scale; PECC = Psychosis Evaluation tool for Common use by Caregivers; SANS—The Scale for the Assessment of Negative Symptoms; SAPS = The Scale for the Assessment of Positive Symptoms; Aerobic Exercise = aerobic exercises interventions; Yoga = yoga training only; HIIT = high intensity interval training; Psychoeducation = psychosocial intervention; exercise = other exercise interventions (e.g., video game exercises intervention); Tai-chi = intervention focused on tai-chi training; Schizophrenia = studies enrolling people with schizophrenia diagnosed; Other psychotic disorders = studies enrolling people with other types of psychotic disorders diagnosed (e.g., schizoaffective disorders and/or other types of psychosis); ^a^ = gender not provided. * positive values of the coefficient mean that a higher level of physical activity is related to better mental health (i.e., less negative symptoms, less positive symptoms, less general psychopathology symptoms.

**Table 2:** Results of the meta-analysis of the relationship between psychotic symptoms and physical activity.

	The Estimate of the Average Effect	Range of Correlation Coefficient (r) Retrieved from Original Studies ^a^		95% CI for the Estimate of the Average Effect	Sample Size	*k*	Heterogeneity		Moderating Effects		*τ*
							*Q*	*I^2^%*	*Q*_b_	*p*	
**Overall effects for all studies included**											
Positive symptoms	0.170 *	−0.510; 0.820		[0.020; 0.312]	884	28	108.169 ***	75.039			0.333
Negative symptoms	0.214 **	−0.450; 0.780		[0.077; 0.343]	1035	31	122.252 ***	75.461			0.325
General psychopathology	0.451 ***	−0.460; 0.890		[0.210; 0.640]	450	19	123.572 ***	85.434			0.542
**Moderator analyses for positive symptoms**											
Moderating effects of the type of PA intervention									1.425	0.700	
Exercises	0.226 *	−0.220; 0.820		[0.026; 0.409]	289	12					0.268
Aerobic exercise	0.291 **	−0.140; 0.570		[0.105; 0.457]	117	5					0.000
Yoga	0.484 *	0.160; 0.670		[0.069; 0.756]	72	3					0.323
HIIT	0.353	−0.010; 0.600		[−0.308; 0.784]	56	2					0.447
Moderating effects of the type of the PA intervention vs. yoga/psychoeducation									1.167	0.280	
Exercises, Aerobic exercises, or HIIT		0.256 **	−0.220; 0.820	[0.107; 0.393]	462	19					0.239
Yoga	0.484 *	0.160; 0.670		[0.069; 0.756]	72	3					0.323
Moderating effects of the type of diagnosis									0.038	0.845	
Schizophrenia	0.182	−0.510; 0.820		[−0.005; 0.356]	579	22					0.376
Other psychotic disorders	0.148	−0.310; 0.600		[−0.146; 0.417]	305	6					0.303
Moderating effects of symptom assessment									3.460	0.063	
PANSS	0.199 *	−0.510; 0.820		[0.024; 0.362]	630	25					0.378
Other measures	−0.056	−0.220; 0.330		[−0.255; 0.148]	254	3					0.118
Moderating effects of the study design									15.184	0.001	
Observational-longitudinal	0.326 ***	−0.140; 0.670		[0.158; 0.475]	252	11					0.178
Experimental-longitudinal	0.250 *	−0.380; 0.820		[0.009; 0.463]	294	12					0.356
Observational-cross-sectional	−0.230	−0.510; −0.008		[−0.437; 0.001]	338	5					0.212
Moderating effects of the study quality									2.613	0.271	
High quality	0.337 *	−0.310; 0.820		[0.077; 0.553]	201	8					0.304
Moderate quality	0.087	−0.510; 0.670		[−0.097; 0.265]	644	17					0.321
Low quality	0.243	−0.140; 0.330		[−0.110; 0.541]	39	3					0.000
**Moderator analyses for negative symptoms**											
Moderating effects of the type of the PA intervention vs. yoga/psychoeducation									7.303	0.121	
Exercises	0.227	−0.450; 0.770		[−0.016; 0.445]	334	13					0.385
Aerobic exercises	0.403 ***	0.160; 0.480		[0.229; 0.552]	117	5					0.000
Yoga	0.358	−0.030; 0.570		[−0.060; 0.669]	72	3					0.298
Psychoeducation	−0.148	−0.360; 0.070		[−0.528; 0.281]	137	2					0.291
HIIT	0.610 *	0.310; 0.780		[0.001; 0.889]	56	2					0.464
Moderating effects of the type of the PA intervention vs. yoga/psychoeducation									0.837	0.360	
Exercises, Aerobic exercises or HIIT		0.315 **	−0.450; 0.770	[0.126; 0.481]	507	20					0.377
Yoga or Psychoeducation		0.130	−0.360; 0.570	[−0.234; 0.461]	209	5					0.369
Moderating effects of the type of diagnosis									0.219	0.640	
Schizophrenia	0.199 *	−0.450; 0.770		[0.030; 0.357]	654	24					0.354
Other psychotic disorders	0.271 *	−0.040; 0.780		[0.008; 0.498]	381	7					0.305
Moderating effects of symptom assessment									0.241	0.624	
PANSS	0.232 **	−0.360; 0.780		[0.076; 0.377]	660	26					0.337
Other	0.142	−0.450; 0.610		[−0.192; 0.447]	375	5					0.351
Moderating effects of the study design									11.935	0.003	
Observational –longitudinal	0.371 ***	−0.060; 0.610		[0.241; 0.488]	373	13					0.150
Experimental-longitudinal	0.257	−0.170; 0.770		[−0.002; 0.483]	294	12					0.393
Observational-cross-sectional	−0.138	−0.450; 0.140		[−0.382; 0.124]	368	6					0.280
Moderating effects of study quality									13.507	0.001.	
High quality	0.392 **	−0.040; 0.780		[0.113; 0.613]	201	8					0.350
Moderate quality	0.088	−0.450; 0.570		[−0.061; 0.233]	750	19					0.259
Low quality	0.523 ***	0.160; 0.610		[0.336; 0.670]	84	4					0.000
**Moderator analyses for general psychopathology symptoms**											
Moderating effect of the type of PA intervention									11.818	0.008	
Exercises	0.420 ***	0.120; 0.880		[0.209; 0.594]	180	9					0.253
Aerobic exercises	0.464 ***	0.170; 0.770		[0.279; 0.615]	95	4					0.000
Yoga	0.795 ***	0.640; 0.820		[0.648; 0.885]	45	2					0.000
HIIT	0.708	0.300; 0.890		[−0.204; 0.962]	56	2					0.756
Moderating effects of the type of the PA intervention vs. yoga/psychoeducation									0.014	0.905	
Exercises, Aerobic exercises or HIIT		0.490 ***	0.120; 0.890	[0.298; 0.644]	331	15					0.365
Yoga or Psychoeducation		0.430	−0.460; 0.820	[−0.646; 0.934]	106	3					1.057
Moderating effects of the type of diagnosis									0.265	.607	
Schizophrenia	0.464 **	−0.460; 0.890		[0.195; 0.669]	401	17					0.582
Other psychotic disorders	0.372 *	0.240; 0.400		[0.091; 0.598]	49	2					0.000
Moderating effects of the study design									0.290	0.590	
Observational-longitudinal	0.468 ***	−0.040; 0.820		[0.264; 0.632]	240	10					0.287
Experimental-longitudinal	0.566 ***	0.180; 0.880		[0.214; 0.788]	149	8					0.540
Moderating effects of the study quality									2.242	0.326	
High quality	0.617 **	0.240; 0.890		[0.302; 0.810]	181	7					0.483
Moderate quality	0.345	−0.460; 0.820		[−0.037; 0.639]	245	10					0.587
Low quality	0.294	0.170; 0.640		[−0.157; 0.644]	24	2					0.000

* *p* < 0.05; ** *p* < 0.01; *** *p* < 0.001; PA = physical activity, PANSS—Positive and Negative Symptoms Scale, HIIT = high intensity interval training; ^a^ = the coefficients from original studies were coded in one direction. Positive values of coefficients are indicating that higher PA levels are related to better mental health (i.e., less positive, negative, and general psychopathology symptoms.)

**Table 3:** The effects of physical activity interventions on psychotic symptoms (randomized control trials or controlled trials with follow-ups).

	Estimates of the Average Effect ^a^	Range of Standardized Difference in Means in Original Studies ^a^	95% CI for the Estimate of the Average Effect	Sample Size	*k*	Heterogeneity		Moderating Effects		*τ*
						*Q*	*I^2^%*	*Q*_b_	*p*	
**Overall effects for all studies included**										
Positive symptoms	0.677 *	−0.295; 3.817	[0.222; 1.132]	283	12	68.033 ***	83.831			0.715
Negative symptoms	0.838 **	−0.0550; 3.826	[0.231; 1.446]	283	12	117.081 ***	90.605			1.006
General psychopathology	1.511 ***	0.337; 5.095	[0.682; 2.340]	138	8	58.586 ***	88.052			1.100
**Analyses for positive symptoms**										
Moderating effects of the type of PA intervention								0.521	0.470	
Exercises	0.743 *	−0.263; 3.817	[0.088; 1.399]	167	7					0.794
Aerobic exercise	1.331	0.640; 2.126	[−0.122; 2.783]	25	2					0.944
Moderating effects of the type of diagnosis								0.113	0.737	
Schizophrenia	0.672 **	−0.295; 3.817	[0.158; 1.187]	234	10					0.744
Other psychotic disorders	0.813 *	0.363; 1.047	[0.176; 1.450]	49	2					0.298
**Analyses for negative symptoms**										
Moderating of effects of PA intervention								3.395	0.065	
Exercises	0.446	−0.550; 3.021	[−0.138; 1.029]	167	7					0.692
Aerobic exercises	1.228 ***	1.030; 1.555	[0.635; 1.820]	25	2					0.000
Moderating effects of the type of diagnosis								0.217	0.641	
Schizophrenia	0.618 **	−0.183; 3.021	[0.173; 1.063]	234	10					0.626
Other psychotic disorders	1.643	−0.550; 3.826	[−2.645; 5.931]	49	2					3.062
**Analyses for** **general psychopathology**										
Moderating effects of the type of PA intervention								0.222	0.637	
Exercises	1.218 *	0.337; 5.095	[0.206; 2.229]	75	5					1.052
Aerobic exercises	1.889	0.615; 3.275	[−0.714; 4.493]	25	2					1.803

*p < 0.05; ** *p* < 0.01; *** *p* < 0.001; PA = physical activity, ^a^ = the coefficients from original studies were coded in one direction. Positive values of coefficients indicate that the higher PA levels are related with better mental health (e.g., less positive or negative symptoms).

## Data Availability

Data affiliated with this study are available at Open Science Framework (see https://osf.io/42cdz/, accessed on 9 May 2022).

## Supplementary Materials

The following supporting information can be downloaded at: https://www.mdpi.com/article/10.3390/jcm11102719/s1, Supplementary Materials 1: Figure S1: Funnel Plot for All Studies Included—Positive Symptoms, Figure S2: Funnel Plot for All Studies Included—Negative Symptoms, Figure S3: Funnel Plot for All Included Studies—General Psychopathology, Figure S4: Funnel Plot for Differences In Means for Positive Symptoms, Figure S5: Funnel Plot for Differences in Means for Negative Symptoms and Figure S6: Funnel Plot for Differences in Means for General Psychopathology; Supplementary Materials 2: Table S1: Overall Effects for all Studies Included—Positive Symptoms, Table S2: Moderating Effects of the Study Design in All Studies Included—Positive Symptoms Table S3: Moderating Effects of the Type of Psychotic Disorders Diagnosis in All Studies Included—Positive Symptoms, Table S4: Moderating Effects of Symptom Assessment in All Studies Included—Positive Symptoms, Table S5: Moderating Effects of the Study Quality in All Studies Included—Positive Symptoms, Table S6: Moderating Effects of the Type of Physical Activity Intervention—Positive Symptoms, Table S7: Moderating Effects of The Type of Physical Activity Intervention (Exercise, Aerobic or HIIT Intervention vs. Yoga)—Positive Symptoms, Table S8: Overall Effects for Randomized Controlled Trials/Controlled Trials—Positive Symptoms, Table S9: Moderating Effects of the Type of Psychotic Disorders Diagnosis in Randomized Controlled Trials/Controlled Trials—Positive Symptoms, Table S10: Moderating Effects of the Type of Physical Activity Intervention in Randomized Controlled Trials/Controlled Trials—Positive Symptoms, Table S11: Overall Effects for All Type of Studies Included—Negative Symptoms, Table S12: Moderating Effects of the Study Design in All Studies Included—Negative Symptoms, Table S13: Moderating Effects of the Type of Diagnosis of Psychotic Disorders in All Studies Included—Negative Symptoms, Table S14: Moderating Effects of Symptom Assessment in All Studies Included—Negative Symptoms, Table S15: Moderating Effects of the Study Quality in All Studies Included—Negative Symptoms, Table S16: Moderating Effects of the Type of Physical Activity Intervention—Negative Symptoms, Table S17: Moderating Effects of the Type of Physical Activity Intervention (Exercise, Aerobic or HIIT Intervention vs. Yoga)– Negative Symptoms, Table S18: Overall Effects for Randomized Controlled Trials/Controlled Trials—Negative Symptoms, Table S19: Moderating Effects of the Type of Diagnosis in Randomized Controlled Trials/Controlled Trials Included—Negative Symptoms, Table S20: Moderating Effects of the Type of Physical Activity Intervention in Randomized Controlled Trials/Controlled Trials—Negative Symptoms, Table S21: Overall Effects for All Type of Studies Included—General Psychopathology, Table S22: Moderating Effects of the Study Design in All Studies Included—General Psychopathology, Table S23: Moderating Effects of the Type of Diagnosis of Psychotic Disorders in All Studies Included—General Psychopathology, Table S24: Moderating Effects of the Study Quality in All Studies Included—General Psychopathology, Table S25: Moderating Effects of the Type of Physical Activity Intervention—General Psychopathology, Table S26: Moderating Effects of the Type of Physical Activity Intervention (Exercise, Aerobic or HIIT Intervention vs. Yoga)—General Psychopathology, Table S27: Overall Effects for All Randomized Controlled Trials/Controlled Trials—General Psychopathology, Table S28: Moderating Effects of the Type of Physical Activity Intervention in Randomized Controlled Trials/Controlled Trials—General Psychopathology.
