# Supplementary material for: Physical Activity, Positive and Negative Symptoms of Psychosis, and General Psychopathology among People with Psychotic Disorders: A Meta-Analysis
Source: J Clin Med. 2022 May 11;11(10):2719. doi: 10.3390/jcm11102719 (PMC9144999; doi:10.3390/jcm11102719)

Physical Activity and Positive Symptoms, Negative Symptoms and General Psychopathology Among People with Psychotic Disorders: a Meta-Analysis

- Figure S1: Funnel Plot for All Studies Included - Positive Symptoms
- Figure S2: Funnel Plot for All Studies Included - Negative Symptoms
- Figure S3: Funnel Plot for All Included Studies - General Psychopathology
- Figure S4: Funnel Plot for Differences In Means for Positive Symptoms
- Figure S5: Funnel Plot for Differences in Means for Negative Symptoms
- Figure S6: Funnel Plot for Differences in Means for General Psychopathology

Figure S1

Funnel Plot for All Studies Included - Positive Symptoms

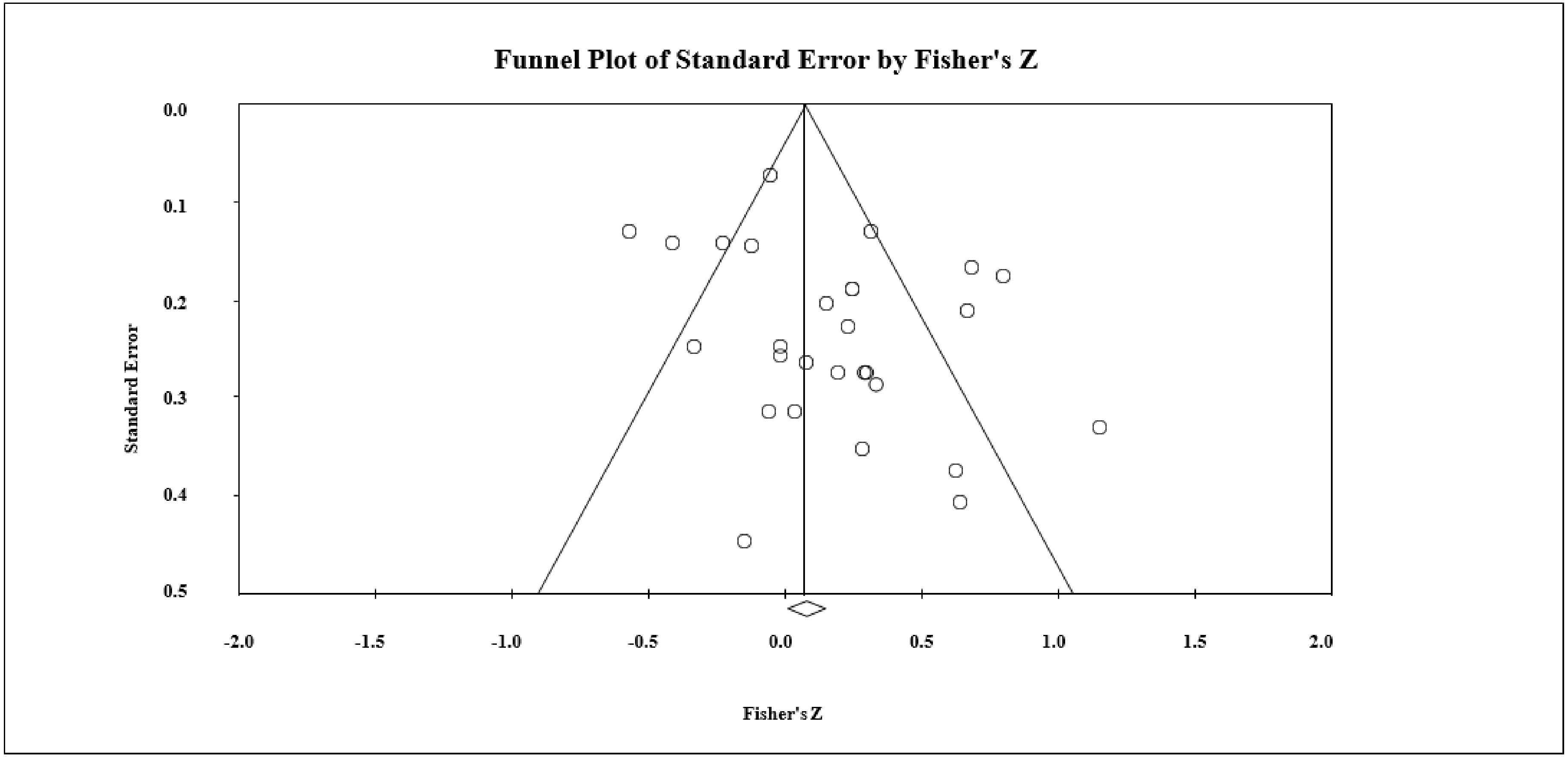

Figure S2

Funnel Plot for All Studies Included - Negative Symptoms

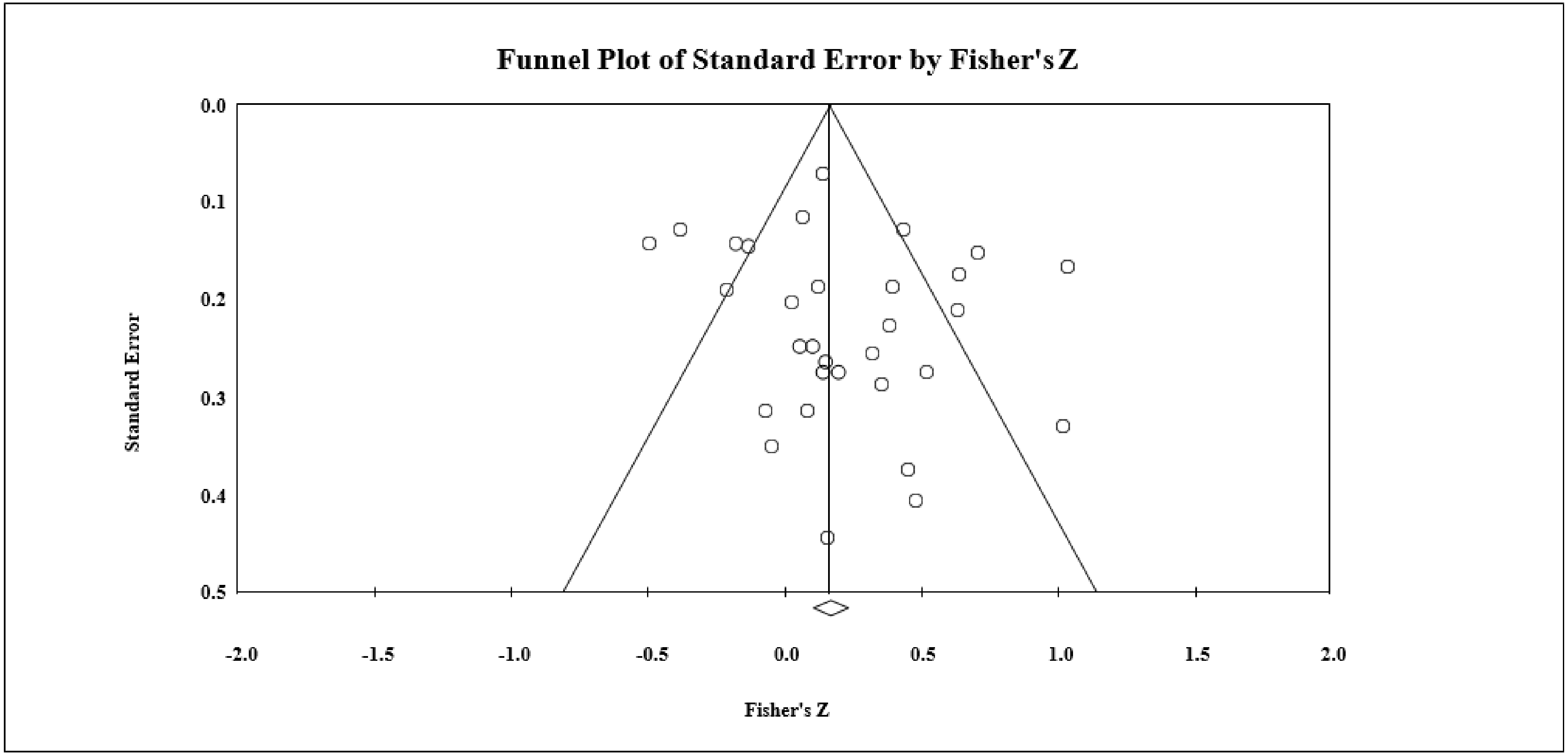

Figure S3

Funnel Plot for All Included Studies - General Psychopathology

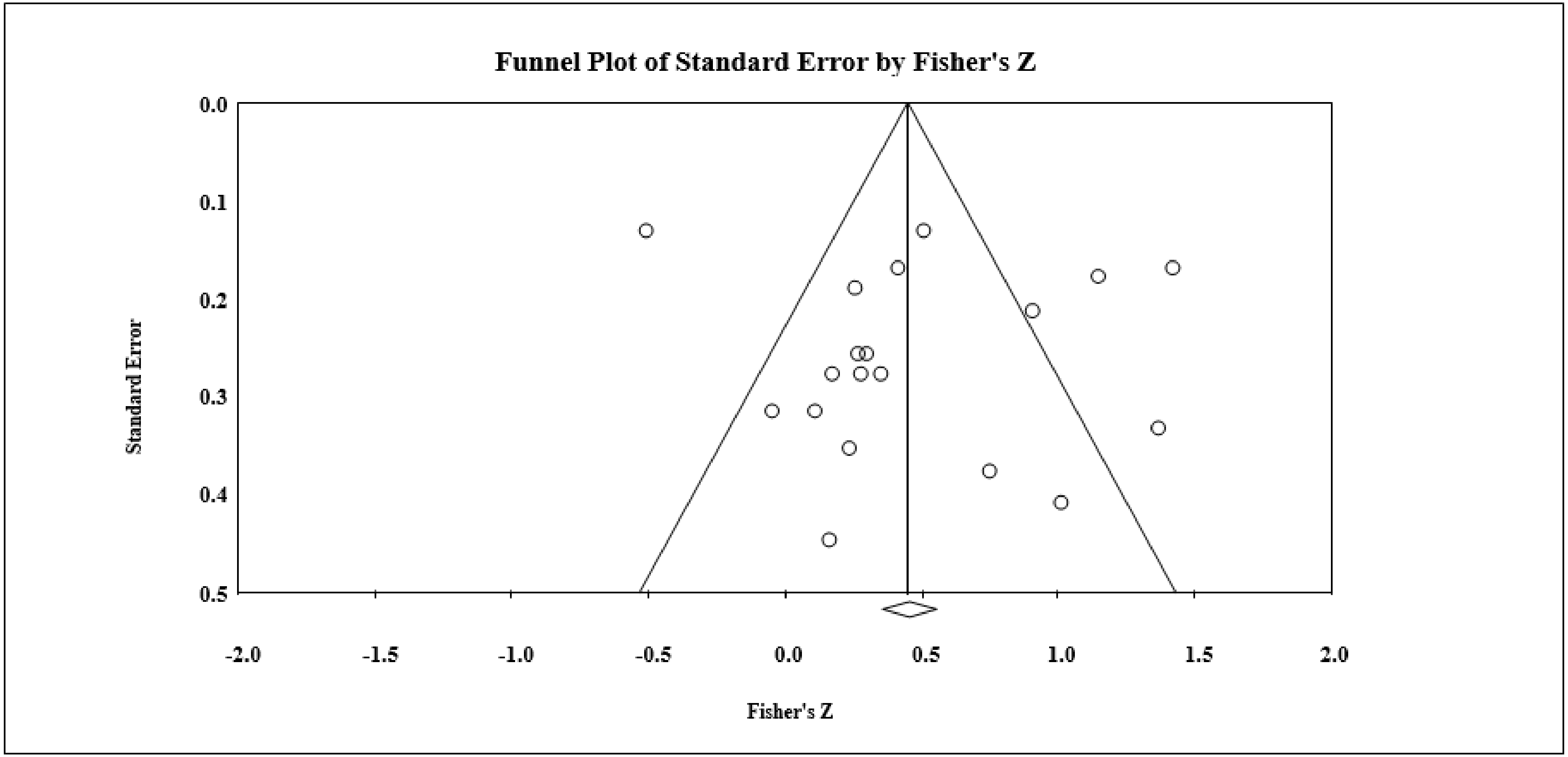

Figure S4

Funnel Plot for Randomized Controlled Trials - Positive Symptoms

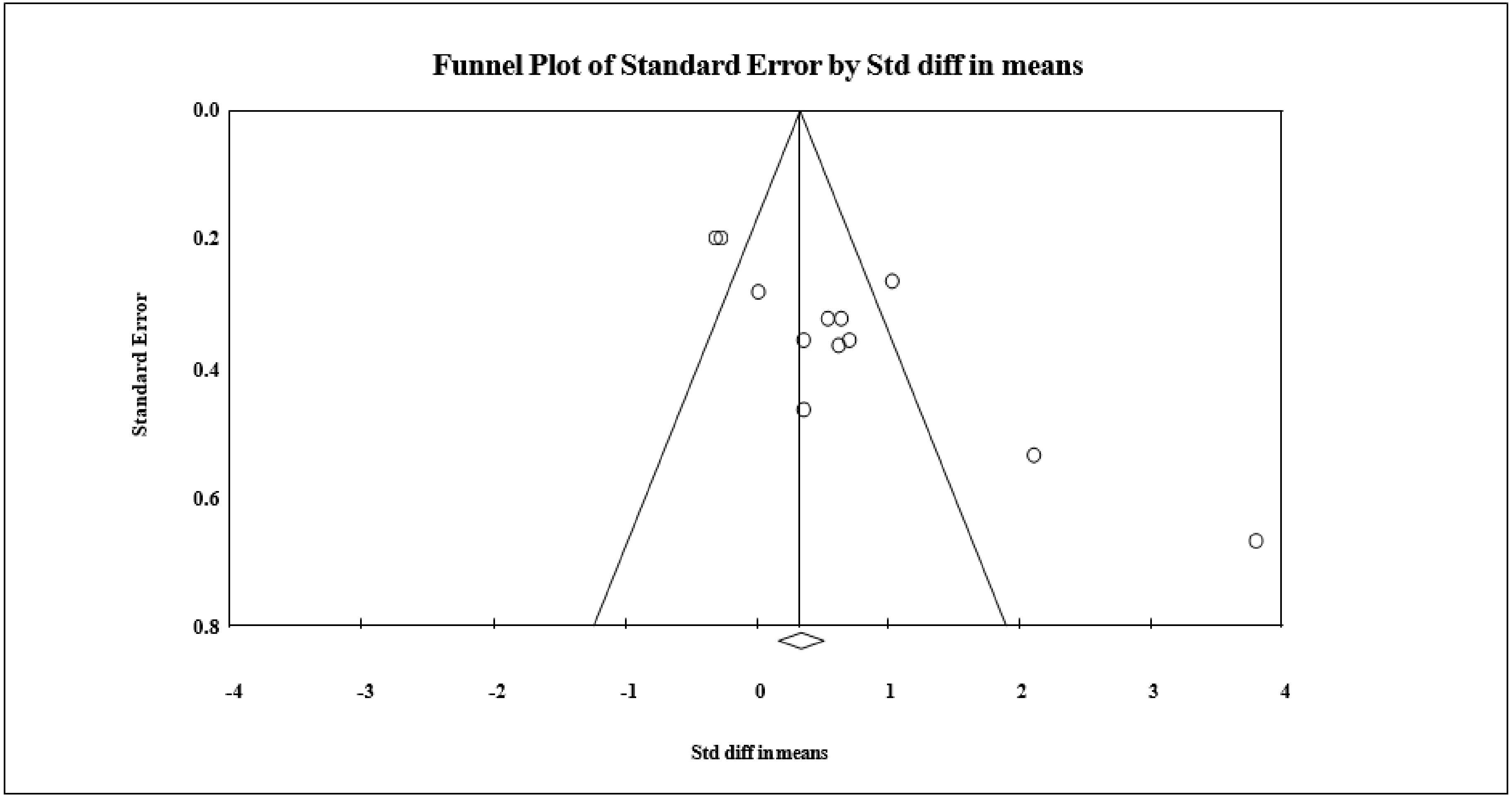

Figure S5

Funnel Plot for Randomized Controlled Trials - Negative Symptoms

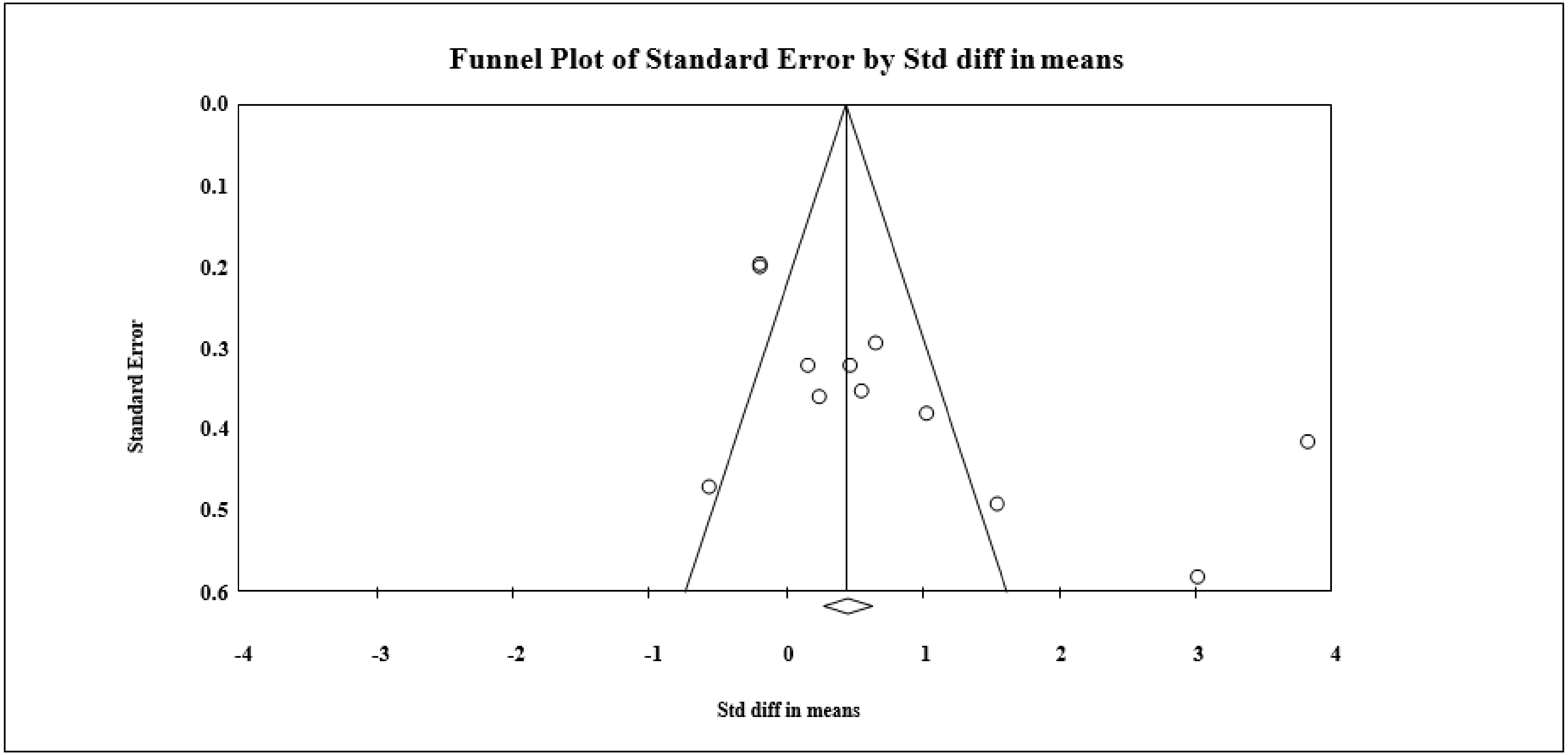

Figure S6

Funnel Plot for Randomized Controlled Trials - General Psychopathology

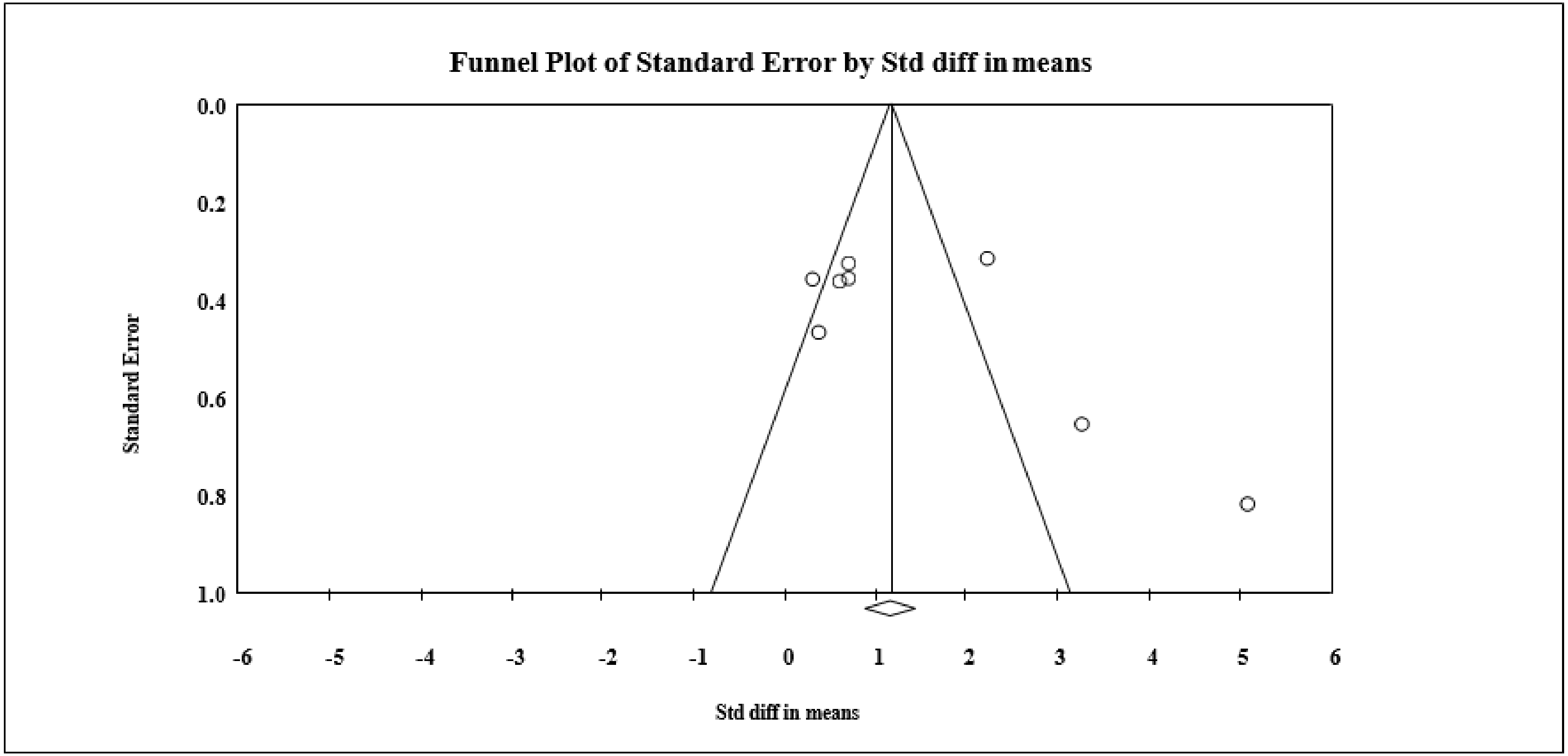

Supplement: Supplementary file 1 [file jcm-11-02719-s001.zip › Supplemental Material 1.pdf]
