# Supplementary material for: Physical Activity, Positive and Negative Symptoms of Psychosis, and General Psychopathology among People with Psychotic Disorders: A Meta-Analysis
Source: J Clin Med. 2022 May 11;11(10):2719. doi: 10.3390/jcm11102719 (PMC9144999; doi:10.3390/jcm11102719)
Supplement: Supplementary file 1 [file jcm-11-02719-s001.zip › Supplemental Material 2.pdf]

Physical Activity and Positive Symptoms, Negative Symptoms and General Psychopathology Among People with Psychotic Disorders: a Meta-Analysis

Supplemental Materials 2

- **Table S1:** Overall Effects for all Studies Included - Positive Symptoms
- **Table S2:** Moderating Effects of the Study Design in All Studies Included – Positive Symptoms
- **Table S3:** Moderating Effects of the Type of Psychotic Disorders Diagnosis in All Studies Included – Positive Symptoms
- **Table S4:** Moderating Effects of Symptom Assessment in All Studies Included – Positive Symptoms
- **Table S5:** Moderating Effects of the Study Quality in All Studies Included – Positive Symptoms
- **Table S6:** Moderating Effects of the Type of Physical Activity Intervention – Positive Symptoms
- **Table S7:** Moderating Effects of Physical Activity Intervention (Exercise, Aerobic or HIIT Intervention vs Yoga) – Positive Symptoms
- **Table S8:** Overall Effects for Randomized Controlled Trials/Controlled Trials - Positive Symptoms
- **Table S9:** Moderating Effects of the Type of Psychotic Disorders Diagnosis in Randomized Controlled Trials/Controlled Trials– Positive Symptoms
- **Table S10:** Moderating Effects of the Type of Physical Activity Intervention in Randomized Controlled Trials Included – Positive Symptoms
- **Table S11:** Overall Effects for All Type of Studies Included - Negative Symptoms

- **Table S12:** Moderating Effects of the Study Design In All Studies Included – Negative Symptoms
- **Table S13:** Moderating Effects of the Type of Diagnosis of Psychotic Disorders in All Studies Included – Negative Symptoms
- **Table S14:** Moderating Effects of Symptom Assessment in All Studies Included – Negative Symptoms
- **Table S15:** Moderating Effects of the Study Quality in All Studies Included – Negative Symptoms
- **Table S16:** Moderating Effects of the Type of Physical Activity Intervention – Negative Symptoms
- **Table S17:** Moderating Effects of the Type of Combined Physical Activity Intervention – Negative Symptoms
- **Table S18:** Overall Effects for Randomized Controlled Trials Included - Negative Symptoms
- **Table S19:** Moderating Effects of the Type of Diagnosis in Randomized Controlled Trials Included - Negative Symptoms
- **Table S20:** Moderating Effects of the Type of Physical Activity Intervention in Randomized Controlled Trials/ Controlled Trials - Negative Symptoms
- **Table S21:** Overall Effects for All Type of Studies Included – General Psychopathology
- **Table S22:** Moderating Effects of the Study Design in all Studies Included – General Psychopathology
- **Table S23:** Moderating Effects of the Type of Diagnosis of Psychotic Disorders in All Studies Included – General Psychopathology

- **Table S24:** Moderating Effects of the Study Quality in All Studies Included – General Psychopathology
- **Table S25:** Moderating Effects of the Type of Physical Activity Intervention – General Psychopathology
- **Table S26:** Moderating Effects of the Type of Physical Activity Intervention *(Exercise, Aerobic or HIIT Intervention vs Yoga)* – General Psychopathology
- **Table S27:** Overall Effects for All Randomized Controlled Trials/ Controlled Trials - General Psychopathology
- **Table S28:** Moderating Effects of the Type of Physical Activity Intervention in Randomized Controlled Trials – General Psychopathology

Table S1

Overall Effects for all Studies Included - Positive Symptoms

Meta Analysis

Studyname	Statistics for each study				
	Correlation	Lower limit	Upper limit	Z-Value	p-Value
Acil et al., 2008	0.330	-0.219	0.720	1.188	0.235
Behere et al., 2011 (exercises)	0.090	-0.408	0.547	0.338	0.736
Behere et al., 2011 (yoga)	0.160	-0.234	0.509	0.791	0.429
Campos et al., 2015	-0.050	-0.585	0.515	-0.158	0.874
Dodd et al., 2011	-0.140	-0.769	0.626	-0.315	0.753
Firth et al., 2018	0.250	-0.114	0.555	1.352	0.177
Fisher et al., 2020	0.280	-0.384	0.753	0.814	0.416
Ho et al., 2016 (exercises)	-0.110	-0.377	0.174	-0.757	0.449
Ho et al., 2016 (Tai-chi)	-0.380	-0.593	-0.117	-2.772	0.006
Kaltsatou et al, 2015	0.200	-0.328	0.633	0.731	0.465
Lee et al., 2013	-0.040	-0.182	0.104	-0.543	0.587
Manjunath et al., 2013 (Exercise)	0.590	0.254	0.799	3.179	0.001
Manjunath et al., 2013 (Yoga)	0.670	0.434	0.820	4.586	0.000
Romain et al., 2019	0.600	0.347	0.772	4.101	0.000
Scheewe et al., 2013	0.250	-0.114	0.555	1.352	0.177
Shimada et al., 2019	0.300	-0.230	0.693	1.116	0.264
Shin et al., 2016	-0.510	-0.675	-0.296	-4.286	0.000
Silva et al., 2015 (aerobic exercises)	0.570	-0.151	0.895	1.586	0.113
Silva et al., 2015 (resistance exercises)	0.820	0.465	0.948	3.470	0.001
Su et al., 2016	0.230	-0.212	0.594	1.021	0.307
Svatkova et al., 2015	0.290	-0.240	0.687	1.076	0.282
Takahashi et al., 2012	0.040	-0.523	0.578	0.127	0.899
Vancampford et al., 2012	-0.220	-0.465	0.056	-1.566	0.117
Vancampfort et al., 2017 (moderate)	-0.008	-0.461	0.448	-0.032	0.974
Vancampfort et al., 2017 (vigorous)	-0.310	-0.670	0.168	-1.282	0.200
Visceglia et al., 2011	0.560	-0.108	0.880	1.674	0.094
Wang et al., 2018	0.310	0.065	0.520	2.462	0.014
Wu et al., 2015	-0.010	-0.475	0.459	-0.039	0.969
	0.170	0.020	0.312	2.212	0.027

Correlation and 95% CI

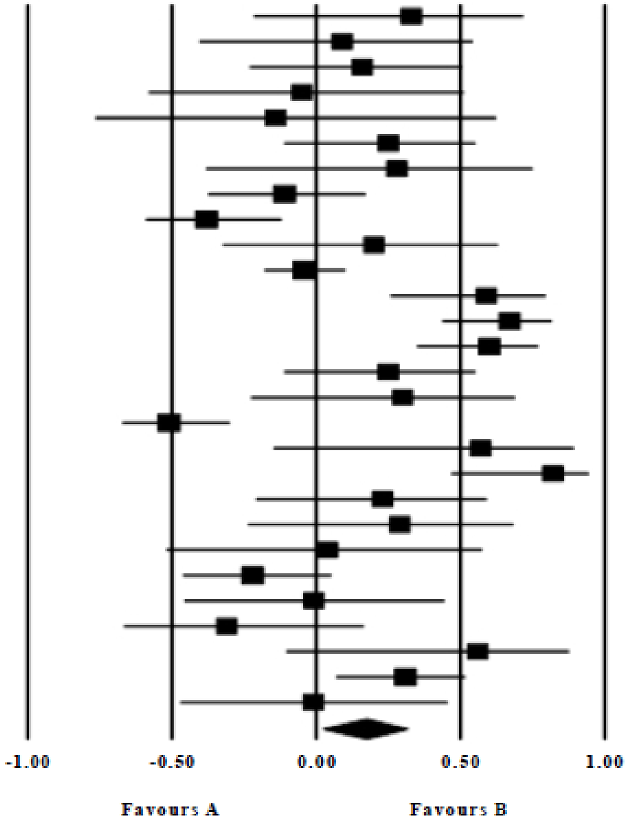

Table S2

Moderating Effects of the Study Design in All Studies Included – Positive Symptoms

Meta Analysis

Group by Study	Study name	Correlation	Lower limit	Upper limit	Z-Value	p-Value
CS	Lee et al., 2013	-0.040	-0.182	0.104	-0.543	0.587
CS	Shin et al., 2016	-0.510	-0.675	-0.296	-4.286	0.000
CS	Vancampford et al., 2012	-0.220	-0.465	0.056	-1.566	0.117
CS	Vancampfort et al., 2017 (moderate)	-0.008	-0.461	0.448	-0.032	0.974
CS	Vancampfort et al., 2017 (vigorous)	-0.310	-0.670	0.168	-1.282	0.200
CS		-0.230	-0.437	0.001	-1.950	0.051
L	Acil et al., 2008	0.330	-0.219	0.720	1.188	0.235
L	Campos et al., 2015	-0.050	-0.585	0.515	-0.158	0.874
L	Dodd et al., 2011	-0.140	-0.769	0.626	-0.315	0.753
L	Firth et al., 2018	0.250	-0.114	0.555	1.352	0.177
L	Manjunath et al., 2013 (Exercise)	0.590	0.254	0.799	3.179	0.001
L	Manjunath et al., 2013 (Yoga)	0.670	0.434	0.820	4.586	0.000
L	Su et al., 2016	0.230	-0.212	0.594	1.021	0.307
L	Takahashi et al., 2012	0.040	-0.523	0.578	0.127	0.899
L	Visceglia et al., 2011	0.560	-0.108	0.880	1.674	0.094
L	Wang et al., 2018	0.310	0.065	0.520	2.462	0.014
L	Wu et al., 2015	-0.010	-0.475	0.459	-0.039	0.969
L		0.326	0.158	0.475	3.707	0.000
L, RCT, EX	Behere et al., 2011 (exercises)	0.090	-0.408	0.547	0.338	0.736
L, RCT, EX	Behere et al., 2011 (yoga)	0.160	-0.234	0.509	0.791	0.429
L, RCT, EX	Fisher et al., 2020	0.280	-0.384	0.753	0.814	0.416
L, RCT, EX	Ho et al., 2016 (exercises)	-0.110	-0.377	0.174	-0.757	0.449
L, RCT, EX	Ho et al., 2016 (Tai-chi)	-0.380	-0.593	-0.117	-2.772	0.006
L, RCT, EX	Kaltsatou et al, 2015	0.200	-0.328	0.633	0.731	0.465
L, RCT, EX	Romain et al., 2019	0.600	0.347	0.772	4.101	0.000
L, RCT, EX	Scheewe et al., 2013	0.250	-0.114	0.555	1.352	0.177
L, RCT, EX	Shimada et al., 2019	0.300	-0.230	0.693	1.116	0.264
L, RCT, EX	Silva et al., 2015 (aerobic exercises)	0.570	-0.151	0.895	1.586	0.113
L, RCT, EX	Silva et al., 2015 (resistance exercises)	0.820	0.465	0.948	3.470	0.001
L, RCT, EX	Svatkova et al., 2015	0.290	-0.240	0.687	1.076	0.282
L, RCT, EX		0.250	0.009	0.463	2.032	0.042
Overall		0.159	0.037	0.276	2.548	0.011

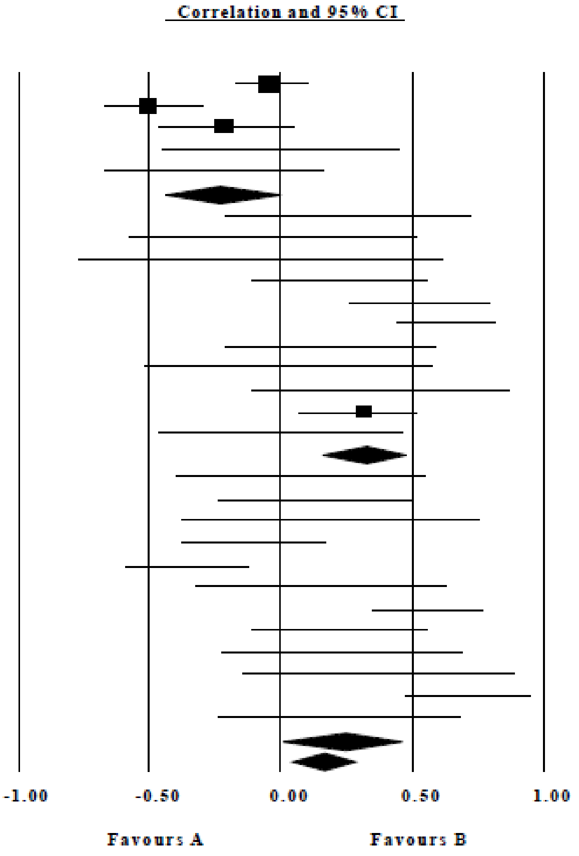

Note: RCT = randomized controlled trials; EX =longitudinal studies with an intervention, no control group; L = observational-longitudinal studies; CS = observational—cross sectional studies.

Table S3

Moderating Effects of the Type of Psychotic Disorders Diagnosis in All Studies Included – Positive Symptoms

Meta Analysis

Group by Diagnosis	Study name	Correlation	Lower limit	Upper limit	Z-Value	p-Value
OPD	Firth et al., 2018	0.250	-0.114	0.555	1.352	0.177
OPD	Fisher et al., 2020	0.280	-0.384	0.753	0.814	0.416
OPD	Lee et al., 2013	-0.040	-0.182	0.104	-0.543	0.587
OPD	Romain et al., 2019	0.600	0.347	0.772	4.101	0.000
OPD	Vancampfort et al., 2017 (moderate)	-0.008	-0.461	0.448	-0.032	0.974
OPD	Vancampfort et al., 2017 (vigorous)	-0.310	-0.670	0.168	-1.282	0.200
OPD		0.148	-0.146	0.417	0.987	0.324
Schizophrenia	Acil et al., 2008	0.330	-0.219	0.720	1.188	0.235
Schizophrenia	Behere et al., 2011 (exercises)	0.090	-0.408	0.547	0.338	0.736
Schizophrenia	Behere et al., 2011 (yoga)	0.160	-0.234	0.509	0.791	0.429
Schizophrenia	Campos et al., 2015	-0.050	-0.585	0.515	-0.158	0.874
Schizophrenia	Dodd et al., 2011	-0.140	-0.769	0.626	-0.315	0.753
Schizophrenia	Ho et al., 2016 (exercises)	-0.110	-0.377	0.174	-0.757	0.449
Schizophrenia	Ho et al., 2016 (Tai-chi)	-0.380	-0.593	-0.117	-2.772	0.006
Schizophrenia	Kaltsatou et al, 2015	0.200	-0.328	0.633	0.731	0.465
Schizophrenia	Manjunath et al., 2013 (Exercise)	0.590	0.254	0.799	3.179	0.001
Schizophrenia	Manjunath et al., 2013 (Yoga)	0.670	0.434	0.820	4.586	0.000
Schizophrenia	Scheewe et al., 2013	0.250	-0.114	0.555	1.352	0.177
Schizophrenia	Shimada et al., 2019	0.300	-0.230	0.693	1.116	0.264
Schizophrenia	Shin et al., 2016	-0.510	-0.675	-0.296	-4.286	0.000
Schizophrenia	Silva et al., 2015 (aerobic exercises)	0.570	-0.151	0.895	1.586	0.113
Schizophrenia	Silva et al., 2015 (resistance exercises)	0.820	0.465	0.948	3.470	0.001
Schizophrenia	Su et al., 2016	0.230	-0.212	0.594	1.021	0.307
Schizophrenia	Svatkova et al., 2015	0.290	-0.240	0.687	1.076	0.282
Schizophrenia	Takahashi et al., 2012	0.040	-0.523	0.578	0.127	0.899
Schizophrenia	Vancampford et al., 2012	-0.220	-0.465	0.056	-1.566	0.117
Schizophrenia	Visceglia et al., 2011	0.560	-0.108	0.880	1.674	0.094
Schizophrenia	Wang et al., 2018	0.310	0.065	0.520	2.462	0.014
Schizophrenia	Wu et al., 2015	-0.010	-0.475	0.459	-0.039	0.969
Schizophrenia		0.182	-0.005	0.356	1.911	0.056
Overall		0.172	0.015	0.321	2.142	0.032

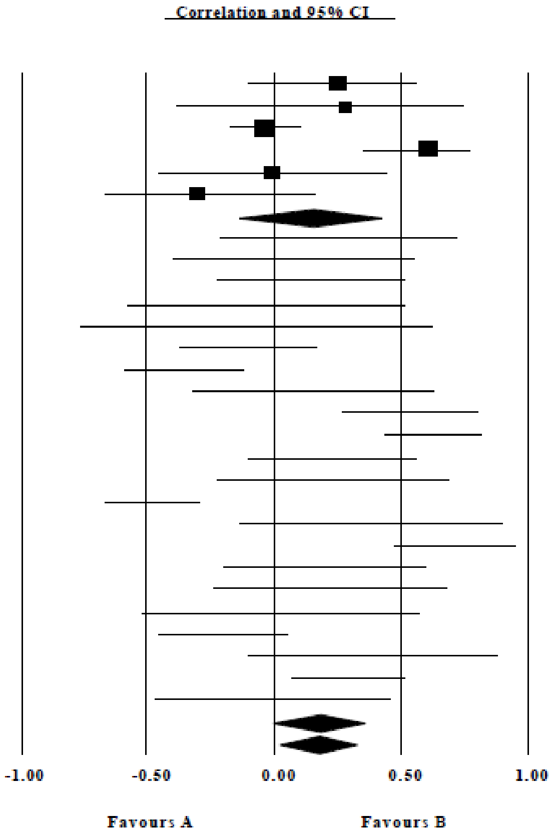

Note: Schizophrenia = studies enrolled people with diagnosed schizophrenia included; OPD = studies enrolled people with other psychotic disorders diagnosed.

Table S4

Moderating Effects of Symptom Assessment in All Studies Included – Positive Symptoms

Meta Analysis

Group by PS Assessment	Study name	Correlation	Lower limit	Upper limit	Z-Value	p-Value
Other	Acil et al., 2008	0.330	-0.219	0.720	1.188	0.235
Other	Lee et al., 2013	-0.040	-0.182	0.104	-0.543	0.587
Other	Vancampford et al., 2012	-0.220	-0.465	0.056	-1.566	0.117
Other		-0.056	-0.255	0.148	-0.532	0.595
PANSS	Behere et al., 2011 (exercises)	0.090	-0.408	0.547	0.338	0.736
PANSS	Behere et al., 2011 (yoga)	0.160	-0.234	0.509	0.791	0.429
PANSS	Campos et al., 2015	-0.050	-0.585	0.515	-0.158	0.874
PANSS	Dodd et al., 2011	-0.140	-0.769	0.626	-0.315	0.753
PANSS	Firth et al., 2018	0.250	-0.114	0.555	1.352	0.177
PANSS	Fisher et al., 2020	0.280	-0.384	0.753	0.814	0.416
PANSS	Ho et al., 2016 (exercises)	-0.110	-0.377	0.174	-0.757	0.449
PANSS	Ho et al., 2016 (Tai-chi)	-0.380	-0.593	-0.117	-2.772	0.006
PANSS	Kaltsatou et al, 2015	0.200	-0.328	0.633	0.731	0.465
PANSS	Manjunath et al., 2013 (Exercise)	0.590	0.254	0.799	3.179	0.001
PANSS	Manjunath et al., 2013 (Yoga)	0.670	0.434	0.820	4.586	0.000
PANSS	Romain et al., 2019	0.600	0.347	0.772	4.101	0.000
PANSS	Scheewe et al., 2013	0.250	-0.114	0.555	1.352	0.177
PANSS	Shimada et al., 2019	0.300	-0.230	0.693	1.116	0.264
PANSS	Shin et al., 2016	-0.510	-0.675	-0.296	-4.286	0.000
PANSS	Silva et al., 2015 (aerobic exercises)	0.570	-0.151	0.895	1.586	0.113
PANSS	Silva et al., 2015 (resistance exercises)	0.820	0.465	0.948	3.470	0.001
PANSS	Su et al., 2016	0.230	-0.212	0.594	1.021	0.307
PANSS	Svatkova et al., 2015	0.290	-0.240	0.687	1.076	0.282
PANSS	Takahashi et al., 2012	0.040	-0.523	0.578	0.127	0.899
PANSS	Vancampfort et al., 2017 (moderate)	-0.008	-0.461	0.448	-0.032	0.974
PANSS	Vancampfort et al., 2017 (vigorous)	-0.310	-0.670	0.168	-1.282	0.200
PANSS	Visceglia et al., 2011	0.560	-0.108	0.880	1.674	0.094
PANSS	Wang et al., 2018	0.310	0.065	0.520	2.462	0.014
PANSS	Wu et al., 2015	-0.010	-0.475	0.459	-0.039	0.969
PANSS		0.199	0.024	0.362	2.227	0.026
Overall		0.091	-0.043	0.222	1.335	0.182

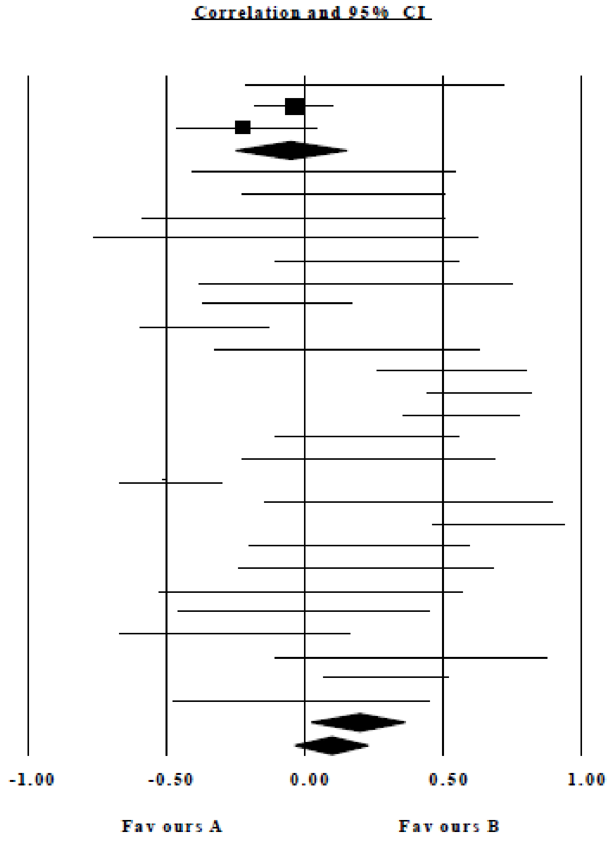

Note: PANSS = Positive and Negative Symptoms Scale; Other = other scales to assess the severity of positive symptoms.

Table S5

Moderating Effects of the Study Quality in All Studies Included – Positive Symptoms

Meta Analysis

Group by Study quality	Study name	Correlation	Lower limit	Upper limit	Z-Value	p-Value
1.00	Fisher et al., 2020	0.280	-0.384	0.753	0.814	0.416
1.00	Romain et al., 2019	0.600	0.347	0.772	4.101	0.000
1.00	Scheewe et al., 2013	0.250	-0.114	0.555	1.352	0.177
1.00	Silva et al., 2015 (aerobic exercises)	0.570	-0.151	0.895	1.586	0.113
1.00	Silva et al., 2015 (resistance exercises)	0.820	0.465	0.948	3.470	0.001
1.00	Vancampfort et al., 2017 (moderate)	-0.008	-0.461	0.448	-0.032	0.974
1.00	Vancampfort et al., 2017 (vigorous)	-0.310	-0.670	0.168	-1.282	0.200
1.00	Wang et al., 2018	0.310	0.065	0.520	2.462	0.014
1.00		0.337	0.077	0.553	2.516	0.012
2.00	Behere et al., 2011 (exercises)	0.090	-0.408	0.547	0.338	0.736
2.00	Behere et al., 2011 (yoga)	0.160	-0.234	0.509	0.791	0.429
2.00	Campos et al., 2015	-0.050	-0.585	0.515	-0.158	0.874
2.00	Firth et al., 2018	0.250	-0.114	0.555	1.352	0.177
2.00	Ho et al., 2016 (exercises)	-0.110	-0.377	0.174	-0.757	0.449
2.00	Ho et al., 2016 (Tai-chi)	-0.380	-0.593	-0.117	-2.772	0.006
2.00	Kaltsatou et al, 2015	0.200	-0.328	0.633	0.731	0.465
2.00	Lee et al., 2013	-0.040	-0.182	0.104	-0.543	0.587
2.00	Manjunath et al., 2013 (Exercise)	0.590	0.254	0.799	3.179	0.001
2.00	Manjunath et al., 2013 (Yoga)	0.670	0.434	0.820	4.586	0.000
2.00	Shin et al., 2016	-0.510	-0.675	-0.296	-4.286	0.000
2.00	Su et al., 2016	0.230	-0.212	0.594	1.021	0.307
2.00	Svatkova et al., 2015	0.290	-0.240	0.687	1.076	0.282
2.00	Takahashi et al., 2012	0.040	-0.523	0.578	0.127	0.899
2.00	Vancampford et al., 2012	-0.220	-0.465	0.056	-1.566	0.117
2.00	Visceglia et al., 2011	0.560	-0.108	0.880	1.674	0.094
2.00	Wu et al., 2015	-0.010	-0.475	0.459	-0.039	0.969
2.00		0.087	-0.097	0.265	0.927	0.354
3.00	Acil et al., 2008	0.330	-0.219	0.720	1.188	0.235
3.00	Dodd et al., 2011	-0.140	-0.769	0.626	-0.315	0.753
3.00	Shimada et al., 2019	0.300	-0.230	0.693	1.116	0.264
3.00		0.243	-0.110	0.541	1.357	0.175
Overall		0.179	0.041	0.311	2.533	0.011

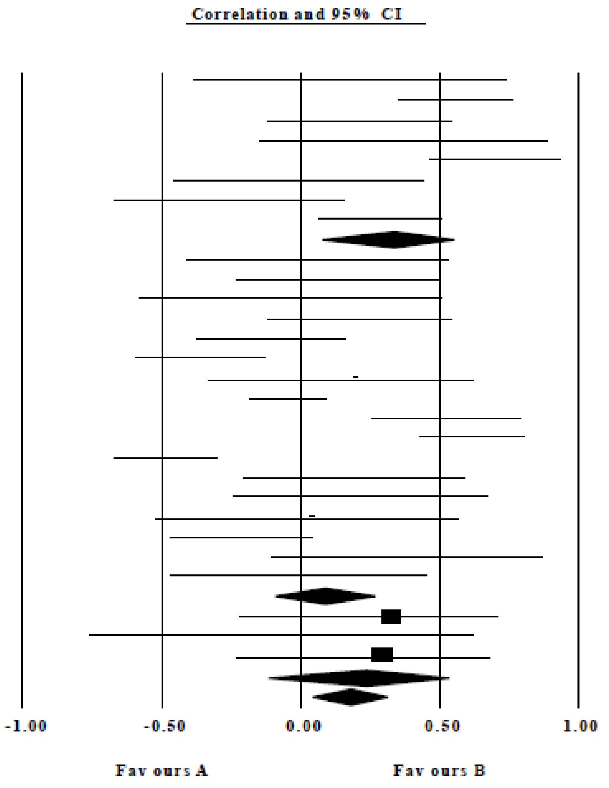

Note: 1 = studies with a high level of quality; 2 = studies with a moderate level of quality; 3 = studies with a low level of quality.

Table S6

Moderating Effects of the Type of Physical Activity Intervention – Positive Symptoms

Meta Analysis

Group by Intervention	Study name	Correlation	Lower limit	Upper limit	Z-Value	p-Value
Aerobic Exercises	Dodd et al., 2011	-0.140	-0.769	0.626	-0.315	0.753
Aerobic Exercises	Shimada et al., 2019	0.300	-0.230	0.693	1.116	0.264
Aerobic Exercises	Silva et al., 2015 (aerobic exercises)	0.570	-0.151	0.895	1.586	0.113
Aerobic Exercises	Su et al., 2016	0.230	-0.212	0.594	1.021	0.307
Aerobic Exercises	Wang et al., 2018	0.310	0.065	0.520	2.462	0.014
Aerobic Exercises		0.291	0.105	0.457	3.026	0.002
Exercises	Acil et al., 2008	0.330	-0.219	0.720	1.188	0.235
Exercises	Behere et al., 2011 (exercises)	0.090	-0.408	0.547	0.338	0.736
Exercises	Firth et al., 2018	0.250	-0.114	0.555	1.352	0.177
Exercises	Fisher et al., 2020	0.280	-0.384	0.753	0.814	0.416
Exercises	Ho et al., 2016 (exercises)	-0.110	-0.377	0.174	-0.757	0.449
Exercises	Kaltsatou et al, 2015	0.200	-0.328	0.633	0.731	0.465
Exercises	Manjunath et al., 2013 (Exercise)	0.590	0.254	0.799	3.179	0.001
Exercises	Scheewe et al., 2013	0.250	-0.114	0.555	1.352	0.177
Exercises	Silva et al., 2015 (resistance exercises)	0.820	0.465	0.948	3.470	0.001
Exercises	Svatkova et al., 2015	0.290	-0.240	0.687	1.076	0.282
Exercises	Takahashi et al., 2012	0.040	-0.523	0.578	0.127	0.899
Exercises	Vancampford et al., 2012	-0.220	-0.465	0.056	-1.566	0.117
Exercises		0.226	0.026	0.409	2.206	0.027
HIIT	Romain et al., 2019	0.600	0.347	0.772	4.101	0.000
HIIT	Wu et al., 2015	-0.010	-0.475	0.459	-0.039	0.969
HIIT		0.353	-0.308	0.784	1.052	0.293
Yoga	Behere et al., 2011 (yoga)	0.160	-0.234	0.509	0.791	0.429
Yoga	Manjunath et al., 2013 (Yoga)	0.670	0.434	0.820	4.586	0.000
Yoga	Visceglia et al., 2011	0.560	-0.108	0.880	1.674	0.094
Yoga		0.484	0.069	0.756	2.256	0.024
Overall		0.284	0.159	0.400	4.335	0.000

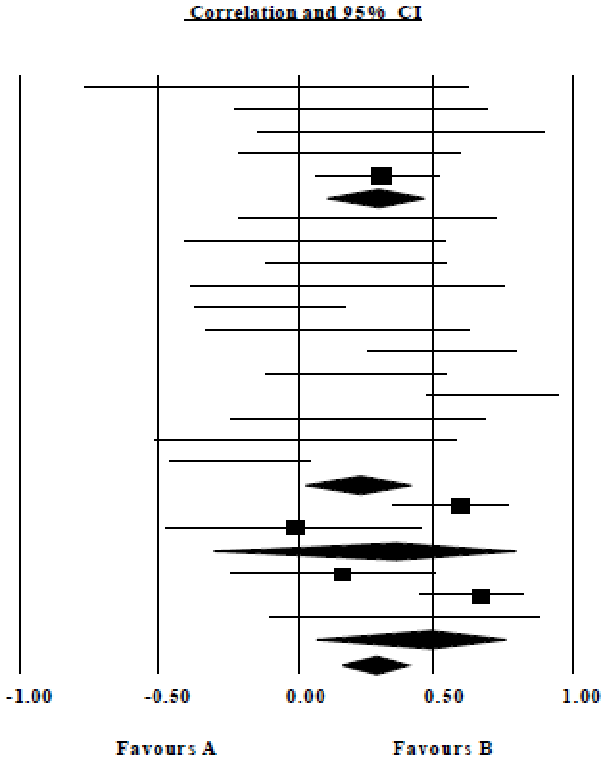

Note: Aerobic Exercise = studies that included aerobic exercises interventions; Exercise = studies that included interventions with various types of exercise, other than aerobic exercise or HIIT (e.g., resistance exercise); HIIT = studies that included high intensity interval training; Yoga = studies that included yoga training only.

Table S7

Moderating Effects of Physical Activity Intervention (Exercise, Aerobic or HIIT Intervention vs Yoga) – Positive Symptoms

Meta Analysis

Group by Intervention	Study name	Correlation	Lower limit	Upper limit	Z-Value	p-Value
A,E,H	Dodd et al., 2011	-0.140	-0.769	0.626	-0.315	0.753
A,E,H	Shimada et al., 2019	0.300	-0.230	0.693	1.116	0.264
A,E,H	Silva et al., 2015 (aerobic exercises)	0.570	-0.151	0.895	1.586	0.113
A,E,H	Su et al., 2016	0.230	-0.212	0.594	1.021	0.307
A,E,H	Wang et al., 2018	0.310	0.065	0.520	2.462	0.014
A,E,H	Acil et al., 2008	0.330	-0.219	0.720	1.188	0.235
A,E,H	Behere et al., 2011 (exercises)	0.090	-0.408	0.547	0.338	0.736
A,E,H	Firth et al., 2018	0.250	-0.114	0.555	1.352	0.177
A,E,H	Fisher et al., 2020	0.280	-0.384	0.753	0.814	0.416
A,E,H	Ho et al., 2016 (exercises)	-0.110	-0.377	0.174	-0.757	0.449
A,E,H	Kaltsatou et al., 2015	0.200	-0.328	0.633	0.731	0.465
A,E,H	Manjunath et al., 2013 (Exercise)	0.590	0.254	0.799	3.179	0.001
A,E,H	Scheewe et al., 2013	0.250	-0.114	0.555	1.352	0.177
A,E,H	Silva et al., 2015 (resistance exercises)	0.820	0.465	0.948	3.470	0.001
A,E,H	Svatkova et al., 2015	0.290	-0.240	0.687	1.076	0.282
A,E,H	Takahashi et al., 2012	0.040	-0.523	0.578	0.127	0.899
A,E,H	Vancampfort et al., 2012	-0.220	-0.465	0.056	-1.566	0.117
A,E,H	Romain et al., 2019	0.600	0.347	0.772	4.101	0.000
A,E,H	Wu et al., 2015	-0.010	-0.475	0.459	-0.039	0.969
A,E,H		0.256	0.107	0.393	3.335	0.001
Yoga	Behere et al., 2011 (yoga)	0.160	-0.234	0.509	0.791	0.429
Yoga	Manjunath et al., 2013 (Yoga)	0.670	0.434	0.820	4.586	0.000
Yoga	Visceglia et al., 2011	0.560	-0.108	0.880	1.674	0.094
Yoga		0.484	0.069	0.756	2.256	0.024
Overall		0.280	0.142	0.409	3.879	0.000

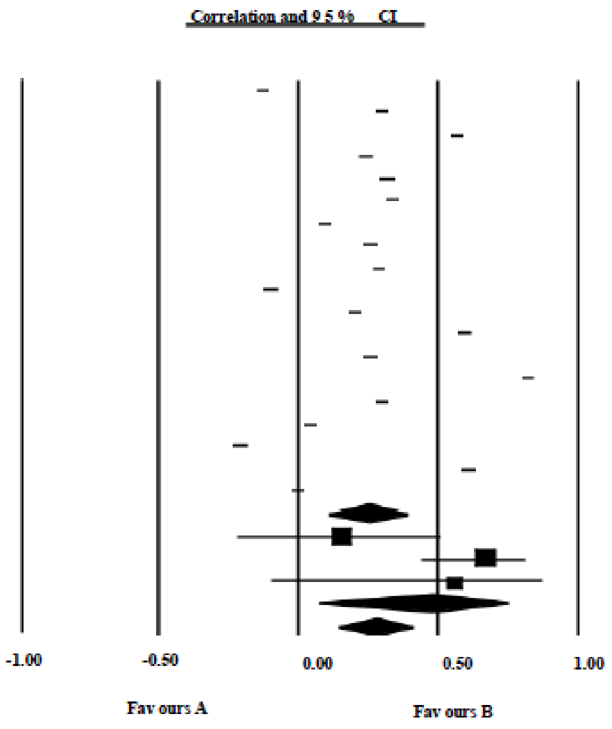

Note: A = Aerobic exercises; E = other exercise interventions; H = High intensity interval training; P = psychoeducation; Y = yoga.

Table S8

Overall Effects for Randomized Controlled Trials/Controlled Trials - Positive Symptoms

Meta Analysis

Study name	Study design	Std diff in means	Standard error	Variance	Lower limit	Upper limit	Z-Value	p-Value
Behere et al., 2011 (Yoga)	Rct	0.018	0.287	0.082	-0.545	0.581	0.063	0.949
Behere et al., 2011 (Exercise)	Rct	0.551	0.329	0.108	-0.094	1.195	1.674	0.094
Fisher et al., 2020	Rct	0.363	0.468	0.219	-0.555	1.281	0.774	0.439
Ho et al., 2016 (Tai - chi)	Rct	-0.295	0.201	0.040	-0.690	0.099	-1.469	0.142
Ho et al., 2016 (Exercise))	Rct	-0.263	0.202	0.041	-0.659	0.133	-1.303	0.192
Kaltsatou et al., 2014	Rct	0.368	0.362	0.131	-0.343	1.078	1.014	0.310
Romain et al., 2019	Rct	1.047	0.268	0.072	0.522	1.572	3.909	0.000
Scheewe et al., 2013	Rct	0.659	0.329	0.108	0.015	1.304	2.005	0.045
Schimada et al., 2019	Rct	0.640	0.368	0.136	-0.083	1.362	1.736	0.083
Silva et al., 2015 (Aerobic exercise)	Rct	2.126	0.539	0.291	1.069	3.183	3.942	0.000
Silva et al., 2015 (Exercise)	Rct	3.817	0.672	0.452	2.500	5.134	5.680	0.000
Svatkova et al., 2015	Rct	0.725	0.360	0.129	0.020	1.430	2.016	0.044
		0.677	0.232	0.054	0.222	1.132	2.917	0.004

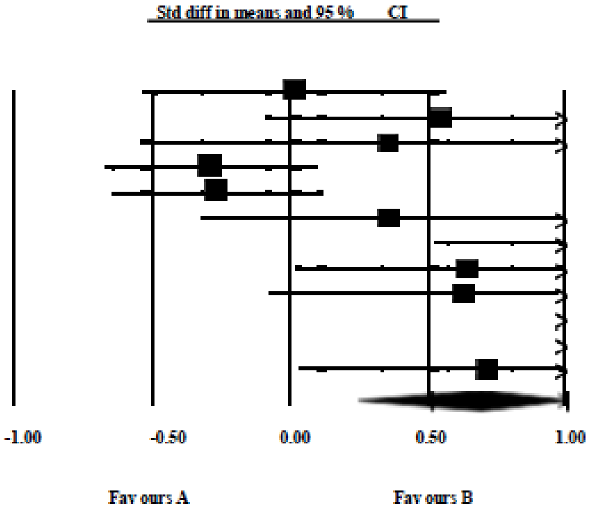

Note: RCT = randomized controlled trials/controlled trials.

Table S9

Moderating Effects of the Type of Psychotic Disorders Diagnosis in Randomized Controlled Trials/Controlled Trials– Positive Symptoms

Meta Analysis

Group by Diagnosis	Study name	Study design	Std diff in means	Standard error	Variance	Lower limit	Upper limit	Z-Value	p-Value
OPD	Fisher et al., 2020	Rct	0.363	0.468	0.219	-0.555	1.281	0.774	0.439
OPD	Romain et al., 2019	Rct	1.047	0.268	0.072	0.522	1.572	3.909	0.000
OPD			0.813	0.325	0.106	0.176	1.450	2.502	0.012
Schizophrenia	Behere et al., 2011 (Yoga)	Rct	0.018	0.287	0.082	-0.545	0.581	0.063	0.949
Schizophrenia	Behere et al., 2011 (Exercise)	Rct	0.551	0.329	0.108	-0.094	1.195	1.674	0.094
Schizophrenia	Ho et al., 2016 (Tai-chi)	Rct	-0.295	0.201	0.040	-0.690	0.099	-1.469	0.142
Schizophrenia	Ho et al., 2016 (Exercise))	Rct	-0.263	0.202	0.041	-0.659	0.133	-1.303	0.192
Schizophrenia	Kaltsatou et al., 2014	Rct	0.368	0.362	0.131	-0.343	1.078	1.014	0.310
Schizophrenia	Scheewe et al., 2013	Rct	0.659	0.329	0.108	0.015	1.304	2.005	0.045
Schizophrenia	Schimada et al., 2019	Rct	0.640	0.368	0.136	-0.083	1.362	1.736	0.083
Schizophrenia	Silva et al., 2015 (Aerobic exercise)	Rct	2.126	0.539	0.291	1.069	3.183	3.942	0.000
Schizophrenia	Silva et al., 2015 (Exercise)	Rct	3.817	0.672	0.452	2.500	5.134	5.680	0.000
Schizophrenia	Svatkova et al., 2015	Rct	0.725	0.360	0.129	0.020	1.430	2.016	0.044
Schizophrenia			0.672	0.262	0.069	0.158	1.187	2.562	0.010
Overall			0.728	0.204	0.042	0.328	1.128	3.565	0.000

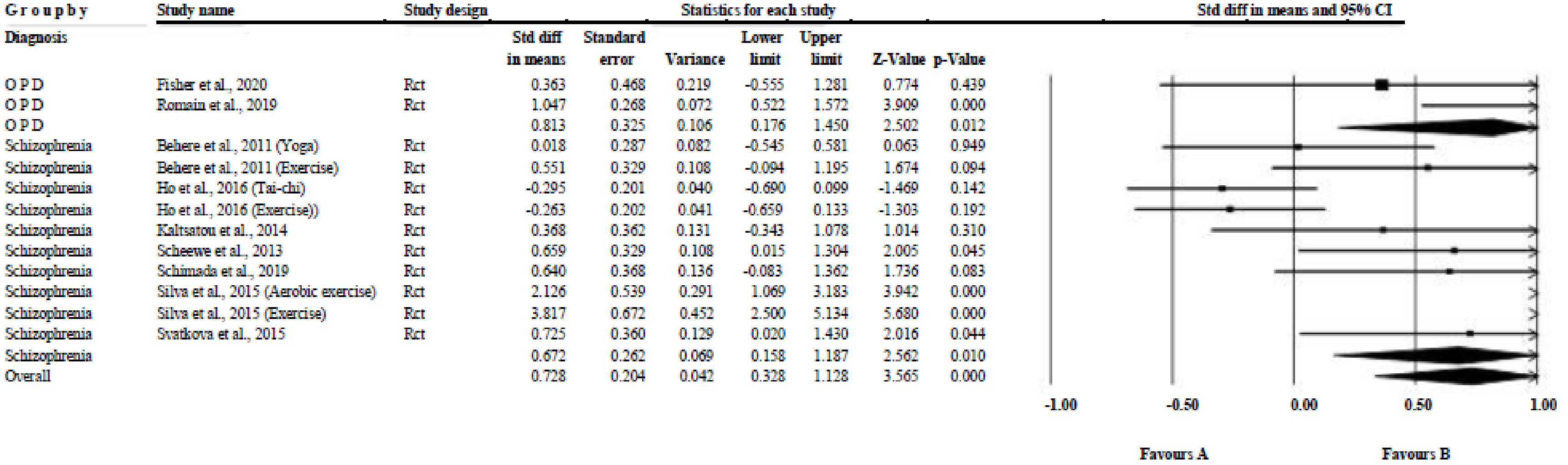

Note: Schizophrenia = studies enrolled people with diagnosed schizophrenia included; OPD = studies enrolled people with other psychotic disorders diagnosed; RCT = randomized controlled trials/controlled trials.

Table S10

Moderating Effects of the Type of Physical Activity Intervention in Randomized Controlled Trials Included – Positive Symptoms

Meta Analysis

Group by Intervention	Study name	Study design	Std diff in means	Standard error	Variance	Lower limit	Upper limit	Z-Value	p-Value
Aerobic Exercises	Schimada et al., 2019	Rct	0.640	0.368	0.136	-0.083	1.362	1.736	0.083
Aerobic Exercises	Silva et al., 2015 (Aerobic exercise)	Rct	2.126	0.539	0.291	1.069	3.183	3.942	0.000
Aerobic Exercises			1.331	0.741	0.549	-0.122	2.783	1.795	0.073
Exercises	Behere et al., 2011 (Exercise)	Rct	0.551	0.329	0.108	-0.094	1.195	1.674	0.094
Exercises	Fisher et al., 2020	Rct	0.363	0.468	0.219	-0.555	1.281	0.774	0.439
Exercises	Ho et al., 2016 (Exercise))	Rct	-0.263	0.202	0.041	-0.659	0.133	-1.303	0.192
Exercises	Kaltsatou et al., 2014	Rct	0.368	0.362	0.131	-0.343	1.078	1.014	0.310
Exercises	Scheewe et al., 2013	Rct	0.659	0.329	0.108	0.015	1.304	2.005	0.045
Exercises	Silva et al., 2015 (Exercise)	Rct	3.817	0.672	0.452	2.500	5.134	5.680	0.000
Exercises	Svatkova et al., 2015	Rct	0.725	0.360	0.129	0.020	1.430	2.016	0.044
Exercises			0.743	0.335	0.112	0.088	1.399	2.222	0.026
Overall			0.843	0.305	0.093	0.245	1.441	2.764	0.006

Std diff in means and 95% CI

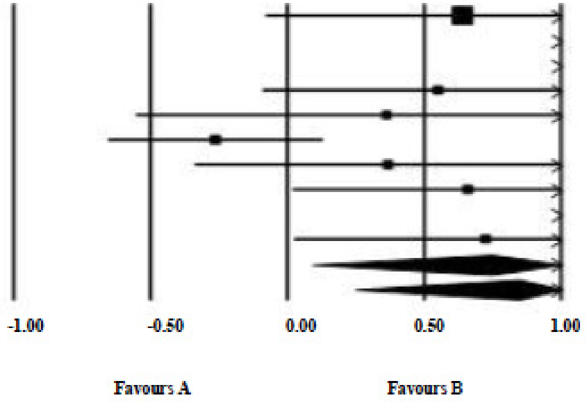

Note: RCT = randomized controlled trials/ controlled trials; Aerobic Exercise = studies with aerobic exercises intervention; Exercises = studies with exercise interventions other than aerobic exercise or HIIT (e.g., resistance training).

Table S11

Overall Effects for All Type of Studies Included - Negative Symptoms

Meta Analysis

Study name	Study design	Correlation	Lower limit	Upper limit	Z-Value	p-Value
Acil et al., 2008	L	0.350	-0.198	0.731	1.266	0.206
Behere et al., 2011 (exercises)	L, RCT, EX	0.150	-0.356	0.588	0.566	0.572
Behere et al., 2011 (Yoga)	L, RCT, EX	0.030	-0.354	0.405	0.147	0.883
Campos et al., 2015	L	-0.060	-0.591	0.508	-0.190	0.849
Dodd et al., 2011	L	0.160	-0.614	0.777	0.361	0.718
Firth et al., 2018	L	0.380	0.030	0.647	2.117	0.034
Fisher et al., 2020	L, RCT, EX	-0.040	-0.625	0.574	-0.113	0.910
Gholipour et al., 2012	L	0.610	0.385	0.766	4.594	0.000
Ho et al., 2016 (exercices)	L, RCT, EX	-0.130	-0.394	0.154	-0.896	0.370
Ho et al., 2016 (Tai-chi)	L, RCT, EX	-0.170	-0.426	0.111	-1.189	0.234
Kaltsatou et al., 2015	L, RCT, EX	0.140	-0.382	0.594	0.508	0.611
Lee et al., 2013	CS	0.140	-0.004	0.278	1.912	0.056
Leutwyler et al., 2013	CS	-0.200	-0.523	0.173	-1.053	0.292
Manjunath et al., 2013 (exercise)	L	0.560	0.212	0.782	2.968	0.003
Matjunath et al., 2013 (yoga)	L	0.570	0.292	0.759	3.663	0.000
Romain et al., 2019	L, RCT, EX	0.780	0.613	0.880	6.184	0.000
Scheewe et al., 2013	L, RCT, EX	0.130	-0.235	0.463	0.692	0.489
Shimada et al., 2019	L, RCT, EX	0.480	-0.021	0.788	1.886	0.059
Shin et al., 2016	CS	-0.360	-0.561	-0.119	-2.870	0.004
Silva et al., 2015 (aerobic)	L, RCT, EX	0.450	-0.305	0.858	1.187	0.235
Silva et al., 2015 (exercises)	L, RCT, EX	0.770	0.351	0.932	3.061	0.002
Su et al., 2016	L	0.370	-0.061	0.685	1.693	0.090
Svatkova et al., 2015	L, RCT, EX	0.200	-0.328	0.633	0.731	0.465
Takahashi et al., 2012	L	0.090	-0.485	0.611	0.285	0.775
Van Citters et al., 2009	L	0.070	-0.158	0.291	0.599	0.549
Vancampford et al., 2012	CS	-0.450	-0.644	-0.202	-3.393	0.001
Vancampfort et al., 2017 (moderate)	CS	0.060	-0.405	0.501	0.240	0.810
Vancampfort et al., 2017 (vigorous)	CS	0.110	-0.362	0.537	0.442	0.659
Visceglia, Lewis, 2011	L	0.430	-0.274	0.834	1.217	0.224
Wang et al., 2018	L	0.410	0.179	0.598	3.346	0.001
Wu et al., 2015	L	0.310	-0.183	0.679	1.241	0.214
		0.214	0.077	0.343	3.044	0.002

Correlation and 95% CI

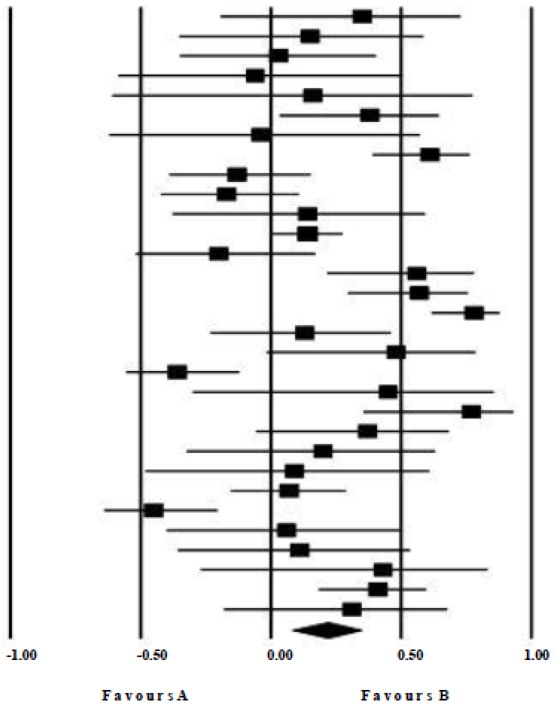

Note: RCT = randomized controlled trials/controlled trials; EX = longitudinal studies with an intervention, no control group; L = observational-longitudinal studies; CS = observational—cross sectional studies.

Table S12

Moderating Effects of the Study Design In All Studies Included – Negative Symptoms

Meta Analysis

Groupby	Studyname	Statistics for each study					Correlationand95% CI
Study design		Correlation	Lower limit	Upper limit	Z-Value	p-Value	
CS	Lee etal.,2013	0.140	-0.004	0.278	1.912	0.056	
CS	Leutwyleretal.,2013	-0.200	-0.523	0.173	-1.053	0.292	
CS	Shin et al.,2016	-0.360	-0.561	-0.119	-2.870	0.004	
CS	Vancampford etal.,2012	-0.450	-0.644	-0.202	-3.393	0.001	
CS	Vancampfort et al., 2017 (moderate)	0.060	-0.405	0.501	0.240	0.810	
CS	Vancampfort et al., 2017 (vigorous)	0.110	-0.362	0.537	0.442	0.659	
CS		-0.138	-0.382	0.124	-1.035	0.301	
L	Acil etal.,2008	0.350	-0.198	0.731	1.266	0.206	
L	Campos et al.,2015	-0.060	-0.591	0.508	-0.190	0.849	
L	Dodd etal.,2011	0.160	-0.614	0.777	0.361	0.718	
L	Firthetal.,2018	0.380	0.030	0.647	2.117	0.034	
L	Gholipouretal.,2012	0.610	0.385	0.766	4.594	0.000	
L	Manjunathetal.,2013 (exercise)	0.560	0.212	0.782	2.968	0.003	
L	Matjunathetal.,2013 (yoga)	0.570	0.292	0.759	3.663	0.000	
L	Su et al.,2016	0.370	-0.061	0.685	1.693	0.090	
L	Takahashi et al.,2012	0.090	-0.485	0.611	0.285	0.775	
L	Van Cittersetal.,2009	0.070	-0.158	0.291	0.599	0.549	
L	Visceglia, Lewis, 2011	0.430	-0.274	0.834	1.217	0.224	
L	Wang etal.,2018	0.410	0.179	0.598	3.346	0.001	
L	Wu etal.,2015	0.310	-0.183	0.679	1.241	0.214	
		0.371	0.241	0.488	5.320	0.000	
RCT, EX, L	Behereetal.,2011 (exercises)	0.150	-0.356	0.588	0.566	0.572	
RCT, EX, L	Behere etal.,2011 (Yoga)	0.030	-0.354	0.405	0.147	0.883	
RCT, EX, L	Fisher et al.,2020	-0.040	-0.625	0.574	-0.113	0.910	
RCT, EX, L	Ho etal.,2016 (exercices)	-0.130	-0.394	0.154	-0.896	0.370	
RCT, EX, L	Ho etal.,2016 (Tai-chi)	-0.170	-0.426	0.111	-1.189	0.234	
RCT, EX, L	Kaltsatou etal.,2015	0.140	-0.382	0.594	0.508	0.611	
RCT, EX, L	Romain et al.,2019	0.780	0.613	0.880	6.184	0.000	
RCT, EX, L	Scheewe et al.,2013	0.130	-0.235	0.463	0.692	0.489	
RCT, EX, L	Shimada et al.,2019	0.480	-0.021	0.788	1.886	0.059	
RCT, EX, L	Silvaetal.,2015 (aerobic)	0.450	-0.305	0.858	1.187	0.235	
RCT, EX, L	Silvaetal.,2015 (exercises)	0.770	0.351	0.932	3.061	0.002	
RCT, EX, L	Svatkovaetal.,2015	0.200	-0.328	0.633	0.731	0.465	
RCT, EX, L		0.257	-0.002	0.483	1.948	0.051	
Overall		0.261	0.153	0.364	4.608	0.000	

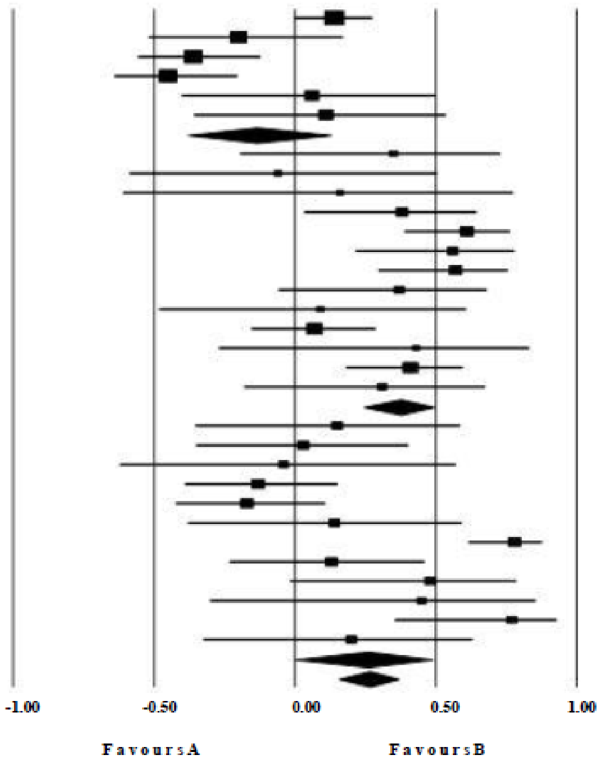

Note: RCT = randomized controlled trials/controlled trials; EX = longitudinal studies with an intervention, no control group; L = observational-longitudinal studies; CS = observational—cross sectional studies.

Table S13

Moderating Effects of the Type of Diagnosis of Psychotic Disorders in All Studies Included – Negative Symptoms

Meta Analysis

Group by Diagnosis	Study name	Correlation	Lower limit	Upper limit	Z-Value	p-Value
OPD	Firth et al., 2018	0.380	0.030	0.647	2.117	0.034
OPD	Fisher et al., 2020	-0.040	-0.625	0.574	-0.113	0.910
OPD	Lee et al., 2013	0.140	-0.004	0.278	1.912	0.056
OPD	Romain et al., 2019	0.780	0.613	0.880	6.184	0.000
OPD	Van Citters et al., 2009	0.070	-0.158	0.291	0.599	0.549
OPD	Vancampfortetal, 2017 (moderate)	0.060	-0.405	0.501	0.240	0.810
OPD	Vancampfortetal, 2017 (vigorous)	0.110	-0.362	0.537	0.442	0.659
OPD		0.271	0.008	0.498	2.021	0.043
Schizophrenia	Acil et al., 2008	0.350	-0.198	0.731	1.266	0.206
Schizophrenia	Behere et al., 2011 (exercises)	0.150	-0.356	0.588	0.566	0.572
Schizophrenia	Behere et al., 2011 (Yoga)	0.030	-0.354	0.405	0.147	0.883
Schizophrenia	Campos et al., 2015	-0.060	-0.591	0.508	-0.190	0.849
Schizophrenia	Dodd et al., 2011	0.160	-0.614	0.777	0.361	0.718
Schizophrenia	Gholipour et al., 2012	0.610	0.385	0.766	4.594	0.000
Schizophrenia	Ho et al., 2016 (exercices)	-0.130	-0.394	0.154	-0.896	0.370
Schizophrenia	Ho et al., 2016 (Tai-chi)	-0.170	-0.426	0.111	-1.189	0.234
Schizophrenia	Kaltsatou et al., 2015	0.140	-0.382	0.594	0.508	0.611
Schizophrenia	Leutwyler et al., 2013	-0.200	-0.523	0.173	-1.053	0.292
Schizophrenia	Manjunathetal., 2013 (exercise)	0.560	0.212	0.782	2.968	0.003
Schizophrenia	Matjunath et al., 2013 (yoga)	0.570	0.292	0.759	3.663	0.000
Schizophrenia	Scheewe et al., 2013	0.130	-0.235	0.463	0.692	0.489
Schizophrenia	Shimada et al., 2019	0.480	-0.021	0.788	1.886	0.059
Schizophrenia	Shin et al., 2016	-0.360	-0.561	-0.119	-2.870	0.004
Schizophrenia	Silva et al., 2015 (aerobic)	0.450	-0.305	0.858	1.187	0.235
Schizophrenia	Silva et al., 2015 (exercises)	0.770	0.351	0.932	3.061	0.002
Schizophrenia	Su et al., 2016	0.370	-0.061	0.685	1.693	0.090
Schizophrenia	Svatkova et al., 2015	0.200	-0.328	0.633	0.731	0.465
Schizophrenia	Takahashi et al., 2012	0.090	-0.485	0.611	0.285	0.775
Schizophrenia	Vancampford et al., 2012	-0.450	-0.644	-0.202	-3.393	0.001
Schizophrenia	Visceglia, Lewis, 2011	0.430	-0.274	0.834	1.217	0.224
Schizophrenia	Wang et al., 2018	0.410	0.179	0.598	3.346	0.001
Schizophrenia	Wu et al., 2015	0.310	-0.183	0.679	1.241	0.214
Schizophrenia		0.199	0.030	0.357	2.303	0.021
Overall		0.220	0.079	0.352	3.028	0.002

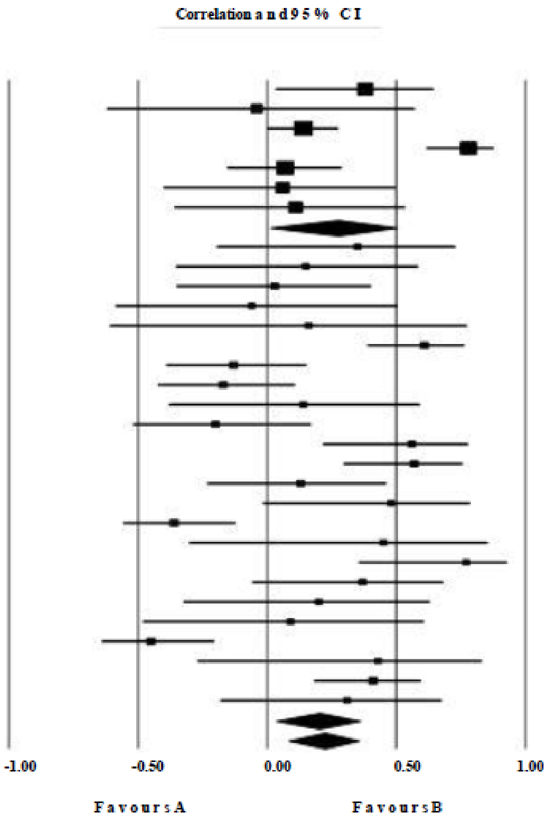

Note: RCT = randomized controlled trials/controlled trials; EX = longitudinal studies with an intervention, no control group; L = observational-longitudinal studies; CS = observational—cross sectional studies; Schizophrenia = studies enrolled people with diagnosed schizophrenia; OPD = studies enrolled people with other psychotic disorders diagnosed.

Table S14

Moderating Effects of Symptom Assessment in All Studies Included – Negative Symptoms

Meta Analysis

Group by PS Assessment	Study name	Correlation	Lower limit	Upper limit	Z-Value	p-Value
Other	Acil et al., 2008	0.350	-0.198	0.731	1.266	0.206
Other	Gholipour et al., 2012	0.610	0.385	0.766	4.594	0.000
Other	Lee et al., 2013	0.140	-0.004	0.278	1.912	0.056
Other	Van Citters et al., 2009	0.070	-0.158	0.291	0.599	0.549
Other	Vancampford et al., 2012	-0.450	-0.644	-0.202	-3.393	0.001
Other		0.142	-0.192	0.447	0.831	0.406
PANSS	Behere et al., 2011 (exercises)	0.150	-0.356	0.588	0.566	0.572
PANSS	Behere et al., 2011 (Yoga)	0.030	-0.354	0.405	0.147	0.883
PANSS	Campos et al., 2015	-0.060	-0.591	0.508	-0.190	0.849
PANSS	Dodd et al., 2011	0.160	-0.614	0.777	0.361	0.718
PANSS	Firth et al., 2018	0.380	0.030	0.647	2.117	0.034
PANSS	Fisher et al., 2020	-0.040	-0.625	0.574	-0.113	0.910
PANSS	Ho et al., 2016 (exercices)	-0.130	-0.394	0.154	-0.896	0.370
PANSS	Ho et al., 2016 (Tai-chi)	-0.170	-0.426	0.111	-1.189	0.234
PANSS	Kaltsatou et al., 2015	0.140	-0.382	0.594	0.508	0.611
PANSS	Leutwyler et al., 2013	-0.200	-0.523	0.173	-1.053	0.292
PANSS	Manjunath et al., 2013 (exercise)L	0.560	0.212	0.782	2.968	0.003
PANSS	Matjunath et al., 2013 (yoga)	0.570	0.292	0.759	3.663	0.000
PANSS	Romain et al., 2019	0.780	0.613	0.880	6.184	0.000
PANSS	Scheewe et al., 2013	0.130	-0.235	0.463	0.692	0.489
PANSS	Shimada et al., 2019	0.480	-0.021	0.788	1.886	0.059
PANSS	Shin et al., 2016	-0.360	-0.561	-0.119	-2.870	0.004
PANSS	Silva et al., 2015 (aerobic)	0.450	-0.305	0.858	1.187	0.235
PANSS	Silva et al., 2015 (exercises)	0.770	0.351	0.932	3.061	0.002
PANSS	Su et al., 2016	0.370	-0.061	0.685	1.693	0.090
PANSS	Svatkova et al., 2015	0.200	-0.328	0.633	0.731	0.465
PANSS	Takahashi et al., 2012	0.090	-0.485	0.611	0.285	0.775
PANSS	Vancampfort et al., 2017 (moderate)	0.060	-0.405	0.501	0.240	0.810
PANSS	Vancampfort et al., 2017 (vigorous)	0.110	-0.362	0.537	0.442	0.659
PANSS	Visceglia, Lewis, 2011	0.430	-0.274	0.834	1.217	0.224
PANSS	Wang et al., 2018	0.410	0.179	0.598	3.346	0.001
PANSS	Wu et al., 2015	0.310	-0.183	0.679	1.241	0.214
PANSS		0.232	0.076	0.377	2.897	0.004
Overall		0.216	0.075	0.349	2.974	0.003

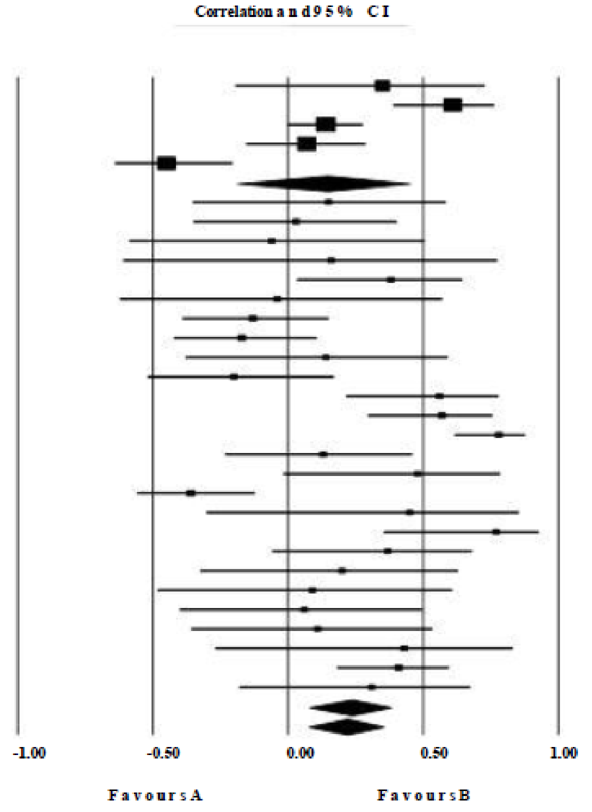

Note: PANSS = Positive and Negative Symptoms Scale; Other = other scales to assess the severity of positive symptoms; RCT = randomized controlled trials/controlled trials; EX = longitudinal studies with an intervention, no control group; L = observational-longitudinal studies; CS = observational—cross sectional studies.

Table S15

Moderating Effects of the Study Quality in All Studies Included – Negative Symptoms

Meta Analysis

Group by Study quality	Study name	Study design	Correlation	Lower limit	Upper limit	Z-Value	p-Value
1.00	Fisheretal., 2020	L, RCT, EX	-0.040	-0.625	0.574	-0.113	0.910
1.00	Romainetal., 2019	L, RCT, EX	0.780	0.613	0.880	6.184	0.000
1.00	Scheeweetal., 2013	L, RCT, EX	0.130	-0.235	0.463	0.692	0.489
1.00	Silva et al., 2015 (aerobic)	L, RCT, EX	0.450	-0.305	0.858	1.187	0.235
1.00	Silva et al., 2015 (exercises)	L, RCT, EX	0.770	0.351	0.932	3.061	0.002
1.00	Vancampfort et al., 2017 (moderate)	CS	0.060	-0.405	0.501	0.240	0.810
1.00	Vancampfort et al., 2017 (vigorous)	CS	0.110	-0.362	0.537	0.442	0.659
1.00	Wangetal., 2018	L	0.410	0.179	0.598	3.346	0.001
1.00			0.392	0.113	0.613	2.705	0.007
2.00	Behere et al., 2011 (exercises)	L, RCT, EX	0.150	-0.356	0.588	0.566	0.572
2.00	Behereetal., 2011 (Yoga)	L, RCT, EX	0.030	-0.354	0.405	0.147	0.883
2.00	Camposetal., 2015	L	-0.060	-0.591	0.508	-0.190	0.849
2.00	Firth et al., 2018	L	0.380	0.030	0.647	2.117	0.034
2.00	Ho et al., 2016 (exercices)	L, RCT, EX	-0.130	-0.394	0.154	-0.896	0.370
2.00	Ho et al., 2016 (Tai - chi)	L, RCT, EX	-0.170	-0.426	0.111	-1.189	0.234
2.00	Kaltsatouetal., 2015	L, RCT, EX	0.140	-0.382	0.594	0.508	0.611
2.00	Leeetal., 2013	CS	0.140	-0.004	0.278	1.912	0.056
2.00	Leutwyler et al., 2013	CS	-0.200	-0.523	0.173	-1.053	0.292
2.00	Manjunath et al., 2013 (exercise)	L	0.560	0.212	0.782	2.968	0.003
2.00	Matjunath et al., 2013 (yoga)	L	0.570	0.292	0.759	3.663	0.000
2.00	Shinetal., 2016	CS	-0.360	-0.561	-0.119	-2.870	0.004
2.00	Suetal., 2016	L	0.370	-0.061	0.685	1.693	0.090
2.00	Svatkovaetal., 2015	L, RCT, EX	0.200	-0.328	0.633	0.731	0.465
2.00	Takahashietal., 2012	L	0.090	-0.485	0.611	0.285	0.775
2.00	Van Citters et al., 2009	L	0.070	-0.158	0.291	0.599	0.549
2.00	Vancampfordetal., 2012	CS	-0.450	-0.644	-0.202	-3.393	0.001
2.00	Visceglia, Lewis, 2011	L	0.430	-0.274	0.834	1.217	0.224
2.00	Wuetal., 2015	L	0.310	-0.183	0.679	1.241	0.214
2.00			0.088	-0.061	0.233	1.161	0.246
3.00	Aciletal., 2008	L	0.350	-0.198	0.731	1.266	0.206
3.00	Doddetal., 2011	L	0.160	-0.614	0.777	0.361	0.718
3.00	Gholipour et al., 2012	L	0.610	0.385	0.766	4.594	0.000
3.00	Shimadaetal., 2019	L, RCT, EX	0.480	-0.021	0.788	1.886	0.059
3.00			0.523	0.336	0.670	4.922	0.000
Overall			0.254	0.143	0.358	4.403	0.000

Correlation and 95% CI

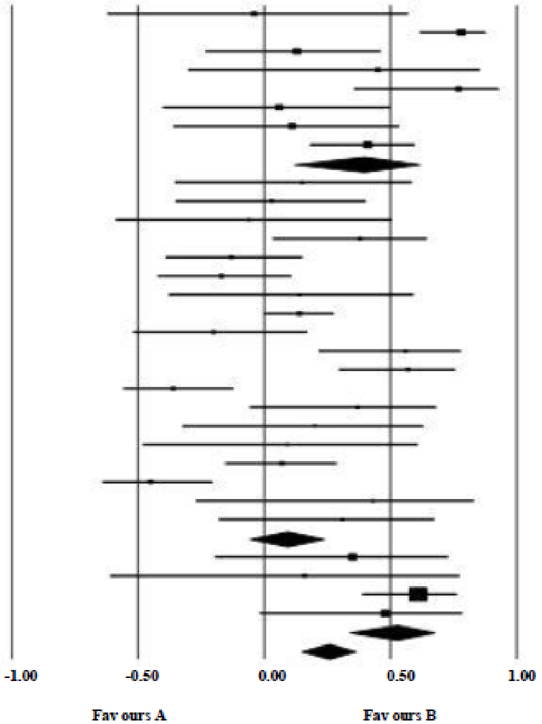

Note: 1 = studies with a high level of quality, 2 = studies with a moderate level of quality, 3 = studies with a low level of quality; RCT = randomized controlled trials/controlled trials; EX = longitudinal studies with an intervention, no control group; L = observational-longitudinal studies; CS = observational—cross sectional studies.

Table S16

Moderating Effects of the Type of Physical Activity Intervention – Negative Symptoms

MetaAnalysis

Groupby Intervention	Studyname	Study design	Correlation	Lower limit	Upper limit	Z-Value	p-Value
AerobicExercises	Doddetal., 2011	L	0.160	-0.614	0.777	0.361	0.718
AerobicExercises	Shimadaetal., 2019	L, RCT, EX	0.480	-0.021	0.788	1.886	0.059
AerobicExercises	Silva et al., 2015 (aerobic)	L, RCT, EX	0.450	-0.305	0.858	1.187	0.235
AerobicExercises	Suetal., 2016	L	0.370	-0.061	0.685	1.693	0.090
AerobicExercises	Wangetal., 2018	L	0.410	0.179	0.598	3.346	0.001
AerobicExercises			0.403	0.229	0.552	4.317	0.000
Exercises	Acil et al., 2008	L	0.350	-0.198	0.731	1.266	0.206
Exercises	Behere et al., 2011 (exercises)L,	RCT, EX	0.150	-0.356	0.588	0.566	0.572
Exercises	Firth et al., 2018	L	0.380	0.030	0.647	2.117	0.034
Exercises	Fisheretal., 2020	L, RCT, EX	-0.040	-0.625	0.574	-0.113	0.910
Exercises	Gholipour et al., 2012	L	0.610	0.385	0.766	4.594	0.000
Exercises	Hoetal., 2016 (exercices)	L, RCT, EX	-0.130	-0.394	0.154	-0.896	0.370
Exercises	Kaltsatouetal., 2015	L, RCT, EX	0.140	-0.382	0.594	0.508	0.611
Exercises	Manjumath et al., 2013 (exercise)	L	0.560	0.212	0.782	2.968	0.003
Exercises	Scheeweetal., 2013	L, RCT, EX	0.130	-0.235	0.463	0.692	0.489
Exercises	Silva et al., 2015 (exercises)	L, RCT, EX	0.770	0.351	0.932	3.061	0.002
Exercises	Svatkova et al., 2015	L, RCT, EX	0.200	-0.328	0.633	0.731	0.465
Exercises	Takahashietal., 2012	L	0.090	-0.485	0.611	0.285	0.775
Exercises	Vancampfordetal., 2012	CS	-0.450	-0.644	-0.202	-3.393	0.001
Exercises			0.227	-0.016	0.445	1.835	0.066
HIIT	Romainetal., 2019	L, RCT, EX	0.780	0.613	0.880	6.184	0.000
HIIT	Wuetal., 2015	L	0.310	-0.183	0.679	1.241	0.214
HIIT			0.610	0.001	0.889	1.962	0.050
Psychoeducation	Shinetal., 2016	CS	-0.360	-0.561	-0.119	-2.870	0.004
Psychoeducation	Van Citters et al., 2009	L	0.070	-0.158	0.291	0.599	0.549
Psychoeducation			-0.148	-0.528	0.281	-0.669	0.504
Yoga	Behereetal., 2011 (Yoga)	L, RCT, EX	0.030	-0.354	0.405	0.147	0.883
Yoga	Matjumath et al., 2013 (yoga)	L	0.570	0.292	0.759	3.663	0.000
Yoga	Visceglia, Lewis, 2011	L	0.430	-0.274	0.834	1.217	0.224
Yoga			0.358	-0.060	0.669	1.689	0.091
Overall			0.310	0.184	0.426	4.674	0.000

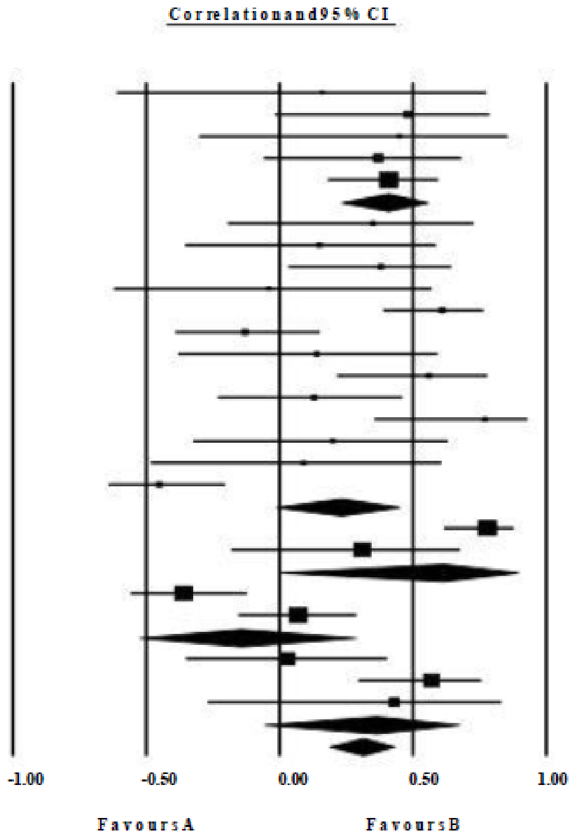

Note: Aerobic Exercises = studies with aerobic exercises interventions; Exercise = studies with interventions with various types of exercise intervention, different from aerobic exercises or HIIT (e.g., resistance exercise); HIIT = studies with high intensity interval training;

Psychoeducation = studies with psychosocial intervention; Yoga = studies with yoga training only; RCT = randomized controlled trials/controlled trials ; EX = longitudinal studies with an intervention, no control group; L = observational-longitudinal studies; CS = observational—cross sectional studies.

Table S17

Moderating Effects of the Type of Combined Physical Activity Intervention – Negative Symptoms

Meta Analysis

Group by Intervention	Study name	Study design	Correlation	Lower limit	Upper limit	Z-Value	p-Value
A,E,H	Dodd et al., 2011	L	0.160	-0.614	0.777	0.361	0.718
A,E,H	Shimada et al., 2019	L, RCT, EX	0.480	-0.021	0.788	1.886	0.059
A,E,H	Silva et al., 2015 (aerobic)	L, RCT, EX	0.450	-0.305	0.858	1.187	0.235
A,E,H	Su et al., 2016	L	0.370	-0.061	0.685	1.693	0.090
A,E,H	Wang et al., 2018	L	0.410	0.179	0.598	3.346	0.001
A,E,H	Acil et al., 2008	L	0.350	-0.198	0.731	1.266	0.206
A,E,H	Behere et al., 2011 (exercises)	L, RCT, EX	0.150	-0.356	0.588	0.566	0.572
A,E,H	Firth et al., 2018	L	0.380	0.030	0.647	2.117	0.034
A,E,H	Fisher et al., 2020	L, RCT, EX	-0.040	-0.625	0.574	-0.113	0.910
A,E,H	Gholipour et al., 2012	L	0.610	0.385	0.766	4.594	0.000
A,E,H	Ho et al., 2016 (exercices)	L, RCT, EX	-0.130	-0.394	0.154	-0.896	0.370
A,E,H	Kaltsatou et al., 2015	L, RCT, EX	0.140	-0.382	0.594	0.508	0.611
A,E,H	Manjunath et al., 2013 (exercise)	L	0.560	0.212	0.782	2.968	0.003
A,E,H	Scheewe et al., 2013	L, RCT, EX	0.130	-0.235	0.463	0.692	0.489
A,E,H	Silva et al., 2015 (exercises)	L, RCT, EX	0.770	0.351	0.932	3.061	0.002
A,E,H	Svatkova et al., 2015	L, RCT, EX	0.200	-0.328	0.633	0.731	0.465
A,E,H	Takahashi et al., 2012	L	0.090	-0.485	0.611	0.285	0.775
A,E,H	Vancampford et al., 2012	CS	-0.450	-0.644	-0.202	-3.393	0.001
A,E,H	Romain et al., 2019	L, RCT, EX	0.780	0.613	0.880	6.184	0.000
A,E,H	Wu et al., 2015	L	0.310	-0.183	0.679	1.241	0.214
A,E,H			0.315	0.126	0.481	3.216	0.001
Y, P	Shin et al., 2016	CS	-0.360	-0.561	-0.119	-2.870	0.004
Y, P	Van Citters et al., 2009	L	0.070	-0.158	0.291	0.599	0.549
Y, P	Behere et al., 2011 (yoga)	L, RCT, EX	0.030	-0.354	0.405	0.147	0.883
Y, P	Matjunath et al., 2013 (yoga)	L	0.570	0.292	0.759	3.663	0.000
Y, P	Visceglia, Lewis, 2011	L	0.430	-0.274	0.834	1.217	0.224
Y, P			0.130	-0.234	0.461	0.694	0.488
Overall			0.274	0.107	0.427	3.160	0.002

Correlation and 95% CI

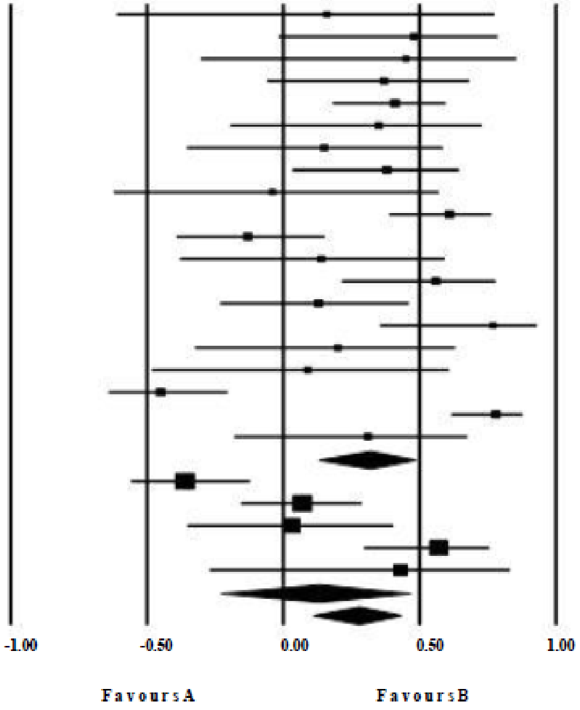

Note: A = Aerobic exercises; E = exercises; H = High intensity interval training; P = psychoeducation; Y = yoga; RCT = randomized controlled trials/controlled trials; EX = longitudinal studies with an intervention, no control group; L = observational-longitudinal studies; CS = observational—cross sectional studies.

Table S18

Overall Effects for Randomized Controlled Trials Included - Negative Symptoms

Meta Analysis

Study name	Std diff in means	Standard error	Variance	Lower limit	Upper limit	Z-Value	p-Value
	Statistics for each study						
Behere et al., 2011 (exercises)	0.177	0.324	0.105	-0.458	0.811	0.546	0.585
Behere et al., 2011 (Yoga)	0.673	0.295	0.087	0.095	1.252	2.282	0.023
Fisher et al., 2020	-0.550	0.473	0.224	-1.478	0.377	-1.163	0.245
Ho et al., 2016 (exercices)	-0.183	0.201	0.041	-0.578	0.212	-0.910	0.363
Ho et al., 2016 (Tai-chi)	-0.174	0.200	0.040	-0.566	0.219	-0.866	0.386
Kaltsatou et al., 2015	0.260	0.361	0.130	-0.447	0.968	0.722	0.471
Romain et al., 2019	3.826	0.419	0.176	3.003	4.648	9.121	0.000
Scheewe et al., 2013	0.485	0.325	0.106	-0.152	1.122	1.493	0.135
Shimada et al., 2019	1.030	0.382	0.146	0.281	1.780	2.694	0.007
Silva et al., 2015 (aerobic)	1.555	0.493	0.243	0.589	2.521	3.154	0.002
Silva et al., 2015 (exercises)	3.021	0.585	0.343	1.874	4.169	5.160	0.000
Svatkova et al., 2015	0.563	0.355	0.126	-0.133	1.259	1.585	0.113
	0.838	0.310	0.096	0.231	1.446	2.703	0.007

Std diff in means and 95% CI

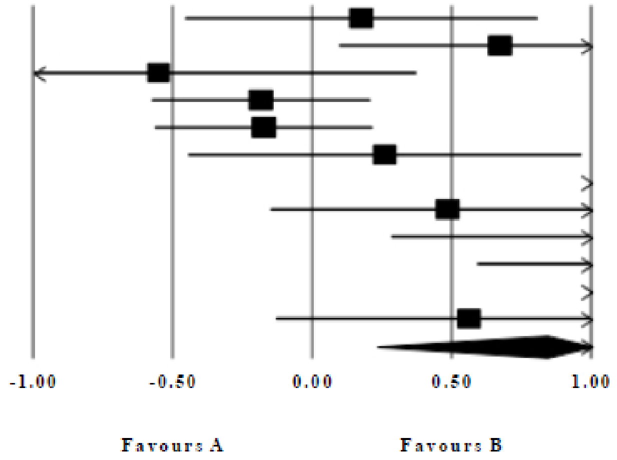

Table S19

Moderating Effects of the Type of Diagnosis in Randomized Controlled Trials Included - Negative Symptoms

Meta Analysis

Group by Diagnosis	Study name	Std diff in means	Standard error	Variance	Lower limit	Upper limit	Z-Value	p-Value
		Statistics for each study						
OPD	Fisheretal.,2020	-0.550	0.473	0.224	-1.478	0.377	-1.163	0.245
OPD	Romainetal.,2019	3.826	0.419	0.176	3.003	4.648	9.121	0.000
OPD		1.643	2.188	4.787	-2.645	5.931	0.751	0.453
Schizophrenia	Behereetal.,2011(exercises)	0.177	0.324	0.105	-0.458	0.811	0.546	0.585
Schizophrenia	Behereetal.,2011(Yoga)	0.673	0.295	0.087	0.095	1.252	2.282	0.023
Schizophrenia	Ho etal.,2016(exercices)	-0.183	0.201	0.041	-0.578	0.212	-0.910	0.363
Schizophrenia	Hoetal.,2016(Tai-chi)	-0.174	0.200	0.040	-0.566	0.219	-0.866	0.386
Schizophrenia	Kaltsatouetal.,2015	0.260	0.361	0.130	-0.447	0.968	0.722	0.471
Schizophrenia	Scheewe etal.,2013	0.485	0.325	0.106	-0.152	1.122	1.493	0.135
Schizophrenia	Shimadaetal.,2019	1.030	0.382	0.146	0.281	1.780	2.694	0.007
Schizophrenia	Silvaetal.,2015(aerobic)	1.555	0.493	0.243	0.589	2.521	3.154	0.002
Schizophrenia	Silvaetal.,2015(exercises)	3.021	0.585	0.343	1.874	4.169	5.160	0.000
Schizophrenia	Svatkovaetal.,2015	0.563	0.355	0.126	-0.133	1.259	1.585	0.113
Schizophrenia		0.618	0.227	0.052	0.173	1.063	2.720	0.007
Overall		0.629	0.226	0.051	0.186	1.071	2.783	0.005

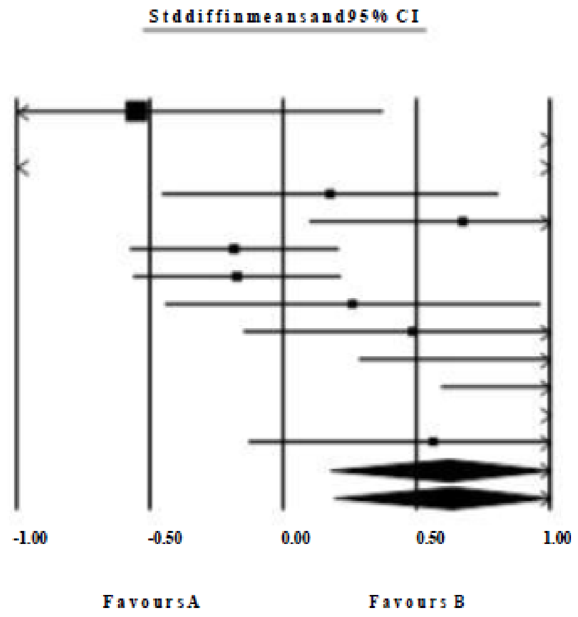

Note: Schizophrenia = studies enrolled people with diagnosed schizophrenia; OPD = studies enrolled people with other psychotic disorders diagnosed; RCT = randomized controlled trials/ controlled trials.

Table S20

Moderating Effects of the Type of Physical Activity Intervention in Randomized Controlled Trials/ Controlled Trials - Negative Symptoms

Meta Analysis

Groupby	Studyname	Statistics for each study						
Intervention		Std diff inmeans	Standard error	Variance	Lower limit	Upper limit	Z-Value	p-Value
AerobicExercises	Shimadaetal., 2019	1.030	0.382	0.146	0.281	1.780	2.694	0.007
AerobicExercises	Silva et al., 2015 (aerobic)	1.555	0.493	0.243	0.589	2.521	3.154	0.002
AerobicExercises		1.228	0.302	0.091	0.635	1.820	4.062	0.000
Exercises	Behere et al., 2011 (exercises)	0.177	0.324	0.105	-0.458	0.811	0.546	0.585
Exercises	Fisheretal., 2020	-0.550	0.473	0.224	-1.478	0.377	-1.163	0.245
Exercises	Hoetal., 2016 (exercices)	-0.183	0.201	0.041	-0.578	0.212	-0.910	0.363
Exercises	Kaltsatouetal., 2015	0.260	0.361	0.130	-0.447	0.968	0.722	0.471
Exercises	Scheeweetal., 2013	0.485	0.325	0.106	-0.152	1.122	1.493	0.135
Exercises	Silvaetal., 2015 (exercises)	3.021	0.585	0.343	1.874	4.169	5.160	0.000
Exercises	Svatkovaetal., 2015	0.563	0.355	0.126	-0.133	1.259	1.585	0.113
Exercises		0.446	0.298	0.089	-0.138	1.029	1.498	0.134
Overall		0.831	0.212	0.045	0.415	1.247	3.918	0.000

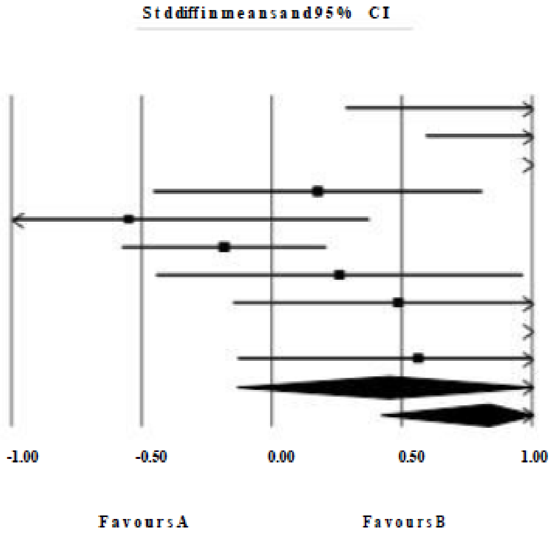

Note: RCT = randomized controlled trial/ controlled trials; Aerobic Exercises = studies with aerobic exercises intervention; Exercises = studies with interventions with various types of exercise intervention, other than HIIT or aerobic exercises (e.g. resistance training).

Table S21

Overall Effects for All Type of Studies Included - General Psychopathology

Meta Analysis

Study name	Study design	Statistics for each study: Correlation	Lower limit	Upper limit	Z-Value	p-Value
Campos et al., 2015	L	-0.040	-0.578	0.523	-0.127	0.899
Dodd et al., 2011	L	0.170	-0.607	0.781	0.384	0.701
Firth et al., 2018	L	0.400	0.092	0.638	2.506	0.012
Fisher et al., 2020	L, RCT, EX	0.240	-0.420	0.734	0.692	0.489
Kaltsatou et al., 2015	L, RCT, EX	0.180	-0.347	0.620	0.656	0.512
Manjunath et al., 2013 (exercise)	L	0.720	0.454	0.868	4.257	0.000
Manjunath et al., 2013 (yoga)	L	0.820	0.670	0.906	6.544	0.000
Romain et al., 2019	L, RCT, EX	0.890	0.797	0.942	8.412	0.000
Scheewe et al., 2013	L, RCT, EX	0.260	-0.104	0.563	1.408	0.159
Scheewe et al., 2013 (2)	L	0.270	-0.225	0.654	1.072	0.284
Shimada et al., 2019	L, RCT, EX	0.340	-0.187	0.715	1.277	0.202
Shin et al., 2016	CS	-0.460	-0.638	-0.235	-3.787	0.000
Silva et al, 2015 (aerobic)	L, RCT, EX	0.770	0.217	0.949	2.499	0.012
Silva et al, 2015 (resistance)	L, RCT, EX	0.880	0.618	0.966	4.127	0.000
Svatkova et al., 2015	L, RCT, EX	0.280	-0.250	0.681	1.037	0.300
Takahashi et al., 2012	L	0.120	-0.461	0.629	0.381	0.703
Visceglia, Lewis, 2011	L	0.640	0.017	0.905	2.006	0.045
Wang et al., 2018	L	0.470	0.250	0.644	3.918	0.000
Wu et al., 2015	L	0.300	-0.194	0.673	1.199	0.231
		0.451	0.210	0.640	3.497	0.000

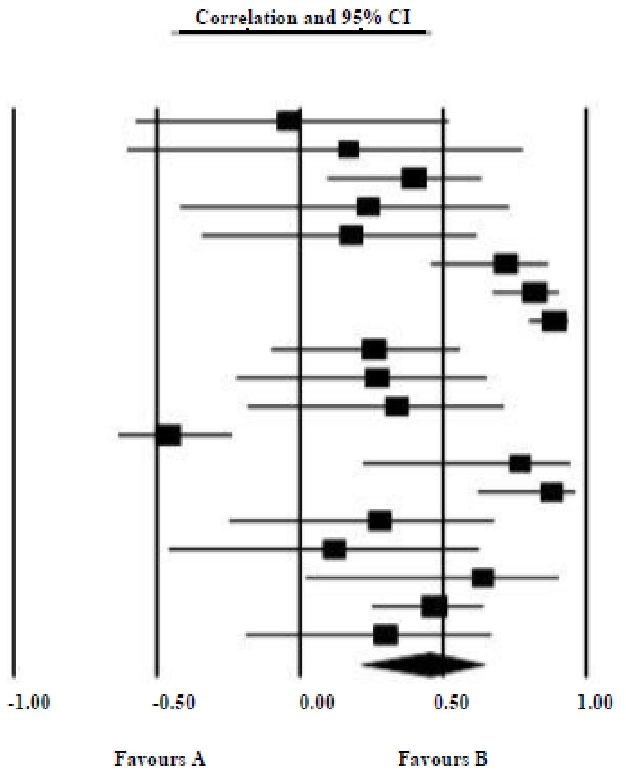

Note: RCT = randomized controlled trials/controlled trials; EX = longitudinal studies with an intervention, no control group; L = observational-longitudinal studies; CS = observational—cross sectional studies.

Table S22

Moderating Effects of the Study Design in all Studies Included – General Psychopathology

Meta Analysis

Group by	Study name	Study design	Correlation	Lower limit	Upper limit	Z-Value	p-Value
Study design							
L	Campos et al., 2015	L	-0.040	-0.578	0.523	-0.127	0.899
L	Dodd et al., 2011	L	0.170	-0.607	0.781	0.384	0.701
L	Firth et al., 2018	L	0.400	0.092	0.638	2.506	0.012
L	Manjunath et al., 2013 (exercise)	L	0.720	0.454	0.868	4.257	0.000
L	Manjunath et al., 2013 (yoga)	L	0.820	0.670	0.906	6.544	0.000
L	Scheewe et al., 2013 (2)	L	0.270	-0.225	0.654	1.072	0.284
L	Takahashi et al., 2012	L	0.120	-0.461	0.629	0.381	0.703
L	Visceglia, Lewis, 2011	L	0.640	0.017	0.905	2.006	0.045
L	Wang et al., 2018	L	0.470	0.250	0.644	3.918	0.000
L	Wu et al., 2015	L	0.300	-0.194	0.673	1.199	0.231
L			0.468	0.264	0.632	4.194	0.000
L, RCT, EX	Fisher et al., 2020	L, RCT, EX	0.240	-0.420	0.734	0.692	0.489
L, RCT, EX	Kaltsatou et al., 2015	L, RCT, EX	0.180	-0.347	0.620	0.656	0.512
L, RCT, EX	Romain et al., 2019	L, RCT, EX	0.890	0.797	0.942	8.412	0.000
L, RCT, EX	Scheewe et al., 2013	L, RCT, EX	0.260	-0.104	0.563	1.408	0.159
L, RCT, EX	Shimada et al., 2019	L, RCT, EX	0.340	-0.187	0.715	1.277	0.202
L, RCT, EX	Silva et al, 2015 (aerobic)	L, RCT, EX	0.770	0.217	0.949	2.499	0.012
L, RCT, EX	Silva et al, 2015 (resistance)	L, RCT, EX	0.880	0.618	0.966	4.127	0.000
L, RCT, EX	Svatkova et al., 2015	L, RCT, EX	0.280	-0.250	0.681	1.037	0.300
L, RCT, EX			0.566	0.214	0.788	2.967	0.003
Overall			0.493	0.321	0.633	5.109	0.000

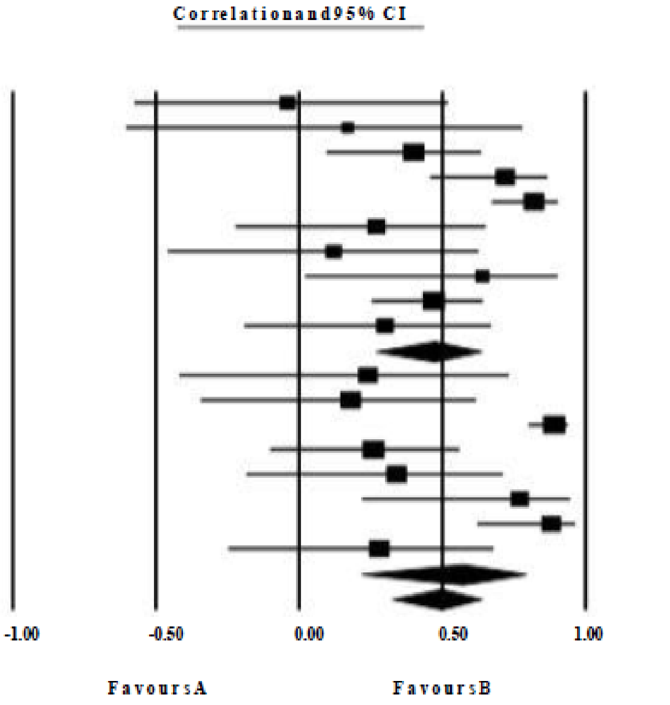

Note: RCT = randomized controlled trials/controlled trials; EX = longitudinal studies with an intervention, no control group; L = observational-longitudinal studies; CS = observational—cross sectional studies.

Table S23

Moderating Effects of the Type of Diagnosis of Psychotic Disorders in All Studies Included – General Psychopathology

Meta Analysis

Group by Diagnosis	Study name	Study design	Correlation	Lower limit	Upper limit	Z-Value	p-Value
OPD	Firth et al., 2018	L	0.400	0.092	0.638	2.506	0.012
OPD	Fisher et al., 2020	L, RCT, EX	0.240	-0.420	0.734	0.692	0.489
OPD			0.372	0.091	0.598	2.560	0.010
Schizophrenia	Campos et al., 2015	L	-0.040	-0.578	0.523	-0.127	0.899
Schizophrenia	Dodd et al., 2011	L	0.170	-0.607	0.781	0.384	0.701
Schizophrenia	Kaltsatou et al., 2015	L, RCT, EX	0.180	-0.347	0.620	0.656	0.512
Schizophrenia	Manjunath et al., 2013 (exercise)	L	0.720	0.454	0.868	4.257	0.000
Schizophrenia	Manjunath et al., 2013 (yoga)	L	0.820	0.670	0.906	6.544	0.000
Schizophrenia	Romain et al., 2019	L, RCT, EX	0.890	0.797	0.942	8.412	0.000
Schizophrenia	Scheewe et al., 2013	L, RCT, EX	0.260	-0.104	0.563	1.408	0.159
Schizophrenia	Scheewe et al., 2013 (2)	L	0.270	-0.225	0.654	1.072	0.284
Schizophrenia	Shimada et al., 2019	L, RCT, EX	0.340	-0.187	0.715	1.277	0.202
Schizophrenia	Shin et al., 2016	CS	-0.460	-0.638	-0.235	-3.787	0.000
Schizophrenia	Silva et al, 2015 (aerobic)	L, RCT, EX	0.770	0.217	0.949	2.499	0.012
Schizophrenia	Silva et al, 2015 (resistance)	L, RCT, EX	0.880	0.618	0.966	4.127	0.000
Schizophrenia	Svatkova et al., 2015	L, RCT, EX	0.280	-0.250	0.681	1.037	0.300
Schizophrenia	Takahashi et al., 2012	L	0.120	-0.461	0.629	0.381	0.703
Schizophrenia	Visceglia, Lewis, 2011	L	0.640	0.017	0.905	2.006	0.045
Schizophrenia	Wang et al., 2018	L	0.470	0.250	0.644	3.918	0.000
Schizophrenia	Wu et al., 2015	L	0.300	-0.194	0.673	1.199	0.231
Schizophrenia			0.464	0.195	0.669	3.225	0.001
Overall			0.418	0.228	0.578	4.085	0.000

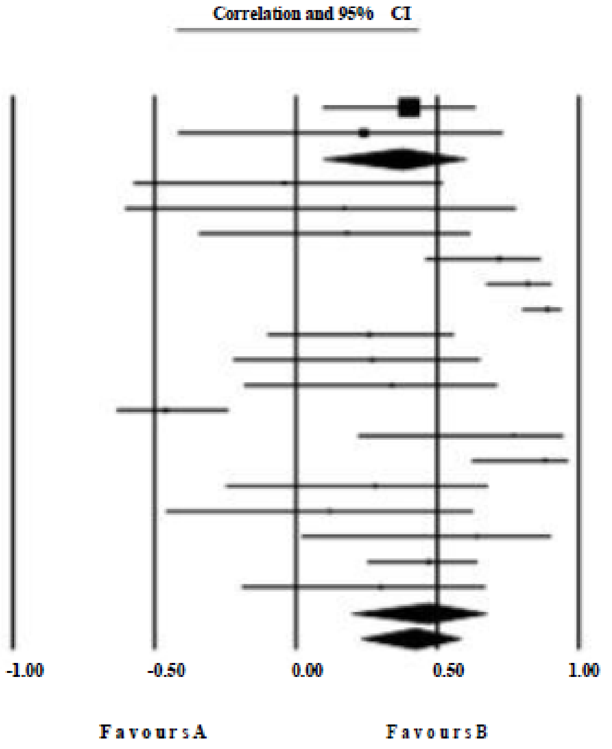

Note: Schizophrenia = studies enrolled people with diagnosed schizophrenia; OPD = studies enrolled people with other psychotic disorders diagnosed; RCT = randomized controlled trials/controlled trials; EX = longitudinal studies with an intervention, no control group; L = observational-longitudinal studies; CS = observational—cross sectional studies.

Table S24

Moderating Effects of the Study Quality in All Studies Included – General Psychopathology

Meta Analysis

Group by Study quality	Study name	Study design	Correlation	Lower limit	Upper limit	Z-Value	p-Value
1.00	Fisher et al., 2020	L, RCT, EX	0.240	-0.420	0.734	0.692	0.489
1.00	Romain et al., 2019	L, RCT, EX	0.890	0.797	0.942	8.412	0.000
1.00	Scheewe et al., 2013	L, RCT, EX	0.260	-0.104	0.563	1.408	0.159
1.00	Scheewe et al., 2013 (2)	L	0.270	-0.225	0.654	1.072	0.284
1.00	Silva et al, 2015 (aerobic)	L, RCT, EX	0.770	0.217	0.949	2.499	0.012
1.00	Silva et al, 2015 (resistance)	L, RCT, EX	0.880	0.618	0.966	4.127	0.000
1.00	Wang et al., 2018	L	0.470	0.250	0.644	3.918	0.000
1.00			0.617	0.302	0.810	3.460	0.001
2.00	Campos et al., 2015	L	-0.040	-0.578	0.523	-0.127	0.899
2.00	Firth et al., 2018	L	0.400	0.092	0.638	2.506	0.012
2.00	Kaltsatou et al., 2015	L, RCT, EX	0.180	-0.347	0.620	0.656	0.512
2.00	Manjunath et al., 2013 (exercise)	L	0.720	0.454	0.868	4.257	0.000
2.00	Manjunath et al., 2013 (yoga)	L	0.820	0.670	0.906	6.544	0.000
2.00	Shin et al., 2016	CS	-0.460	-0.638	-0.235	-3.787	0.000
2.00	Svatkova et al., 2015	L, RCT, EX	0.280	-0.250	0.681	1.037	0.300
2.00	Takahashi et al., 2012	L	0.120	-0.461	0.629	0.381	0.703
2.00	Visceglia, Lewis, 2011	L	0.640	0.017	0.905	2.006	0.045
2.00	Wu et al., 2015	L	0.300	-0.194	0.673	1.199	0.231
2.00			0.345	-0.037	0.639	1.775	0.076
3.00	Dodd et al., 2011	L	0.170	-0.607	0.781	0.384	0.701
3.00	Shimada et al., 2019	L, RCT, EX	0.340	-0.187	0.715	1.277	0.202
3.00			0.294	-0.157	0.644	1.287	0.198
Overall			0.439	0.225	0.613	3.812	0.000

Correlation and 95% CI

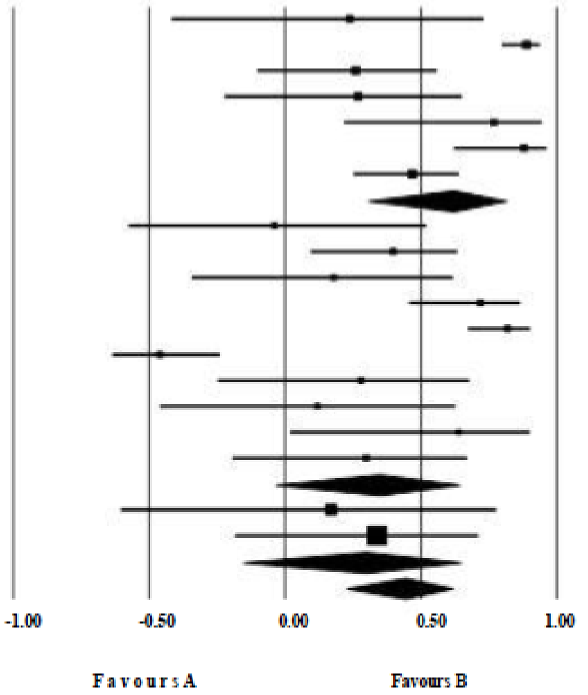

Note: 1 = studies with a high level of quality, 2 = studies with a moderate level of quality, 3 = studies with a high level of quality; RCT = randomized controlled trials/controlled trials; EX = longitudinal studies with an intervention, no control group; L = observational-longitudinal studies; CS = observational—cross sectional studies.

Table S25

Moderating Effects of the Type of Physical Activity Intervention – General Psychopathology

Meta Analysis

Group by Intervention	Study name	Study design	Correlation	Lower limit	Upper limit	Z-Value	p-Value
Aerobic Exercises	Dodd et al., 2011	L	0.170	-0.607	0.781	0.384	0.701
Aerobic Exercises	Shimada et al., 2019	L, RCT, EX	0.340	-0.187	0.715	1.277	0.202
Aerobic Exercises	Silva et al, 2015 (aerobic)	L, RCT, EX	0.770	0.217	0.949	2.499	0.012
Aerobic Exercises	Wang et al., 2018	L	0.470	0.250	0.644	3.918	0.000
Aerobic Exercises			0.464	0.279	0.615	4.575	0.000
Exercises	Firth et al., 2018	L	0.400	0.092	0.638	2.506	0.012
Exercises	Fisher et al., 2020	L, RCT, EX	0.240	-0.420	0.734	0.692	0.489
Exercises	Kaltsatou et al., 2015	L, RCT, EX	0.180	-0.347	0.620	0.656	0.512
Exercises	Manjunath et al., 2013 (exercise)	L	0.720	0.454	0.868	4.257	0.000
Exercises	Scheewe et al., 2013	L, RCT, EX	0.260	-0.104	0.563	1.408	0.159
Exercises	Scheewe et al., 2013 (2)	L	0.270	-0.225	0.654	1.072	0.284
Exercises	Silva et al, 2015 (resistance)	L, RCT, EX	0.880	0.618	0.966	4.127	0.000
Exercises	Svatkova et al., 2015	L, RCT, EX	0.280	-0.250	0.681	1.037	0.300
Exercises	Takahashi et al., 2012	L	0.120	-0.461	0.629	0.381	0.703
Exercises			0.420	0.209	0.594	3.717	0.000
HIIT	Romain et al., 2019	L, RCT, EX	0.890	0.797	0.942	8.412	0.000
HIIT	Wu et al., 2015	L	0.300	-0.194	0.673	1.199	0.231
HIIT			0.708	-0.204	0.962	1.588	0.112
Yoga	Manjunath et al., 2013 (yoga)	L	0.820	0.670	0.906	6.544	0.000
Yoga	Visceglia, Lewis, 2011	L	0.640	0.017	0.905	2.006	0.045
Yoga			0.795	0.648	0.885	6.777	0.000
Overall			0.542	0.435	0.633	8.449	0.000

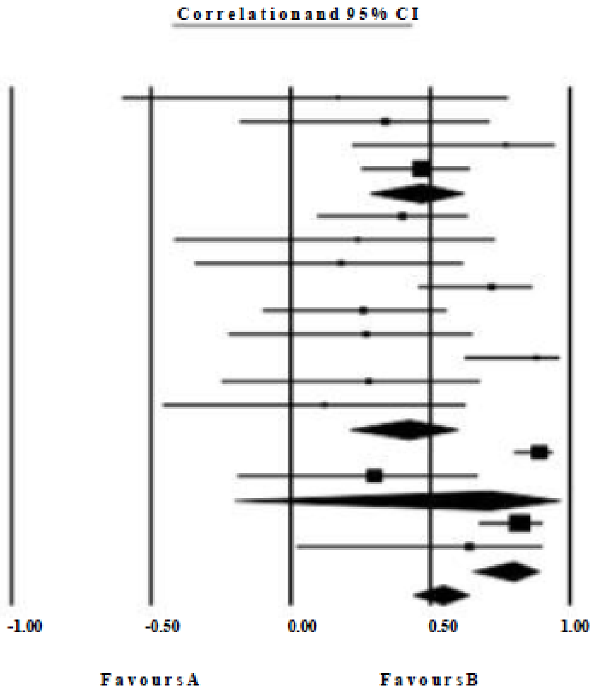

Note: Aerobic Exercises = studies included aerobic exercises interventions; Exercise = studies included interventions with various types of exercises (e.g., resistance exercise); HIIT = studies enrolled high intensity interval training; Yoga = studies enrolled yoga training only; RCT = randomized controlled trials/controlled trials; EX = longitudinal studies with an intervention, no control group; L = observational-longitudinal studies; CS = observational—cross sectional studies.

Table S26

Moderating Effects of Combined Physical Activity Intervention – General Psychopathology

Meta Analysis

Group by Intervention	Study name	Study design	Correlation	Lower limit	Upper limit	Z-Value	p-Value
A,E,H	Dodd et al., 2011	L	0.170	-0.607	0.781	0.384	0.701
A,E,H	Shimada et al., 2019	L, RCT, EX	0.340	-0.187	0.715	1.277	0.202
A,E,H	Silva et al, 2015 (aerobic)	L, RCT, EX	0.770	0.217	0.949	2.499	0.012
A,E,H	Wang et al., 2018	L	0.470	0.250	0.644	3.918	0.000
A,E,H	Firth et al., 2018	L	0.400	0.092	0.638	2.506	0.012
A,E,H	Fisher et al., 2020	L, RCT, EX	0.240	-0.420	0.734	0.692	0.489
A,E,H	Kaltsatou et al., 2015	L, RCT, EX	0.180	-0.347	0.620	0.656	0.512
A,E,H	Manjunath et al., 2013 (exercise)	L	0.720	0.454	0.868	4.257	0.000
A,E,H	Scheewe et al., 2013	L, RCT, EX	0.260	-0.104	0.563	1.408	0.159
A,E,H	Scheewe et al., 2013 (2)	L	0.270	-0.225	0.654	1.072	0.284
A,E,H	Silva et al, 2015 (resistance)	L, RCT, EX	0.880	0.618	0.966	4.127	0.000
A,E,H	Svatkova et al., 2015	L, RCT, EX	0.280	-0.250	0.681	1.037	0.300
A,E,H	Takahashi et al., 2012	L	0.120	-0.461	0.629	0.381	0.703
A,E,H	Romain et al., 2019	L, RCT, EX	0.890	0.797	0.942	8.412	0.000
A,E,H	Wu et al., 2015	L	0.300	-0.194	0.673	1.199	0.231
A,E,H			0.490	0.298	0.644	4.594	0.000
P, Y	Shin et al., 2016	CS	-0.460	-0.638	-0.235	-3.787	0.000
P, Y	Manjunath et al., 2013 (yoga)	L	0.820	0.670	0.906	6.544	0.000
P, Y	Visceglia, Lewis, 2011	L	0.640	0.017	0.905	2.006	0.045
P, Y			0.430	-0.646	0.934	0.733	0.463
Overall			0.488	0.299	0.640	4.650	0.000

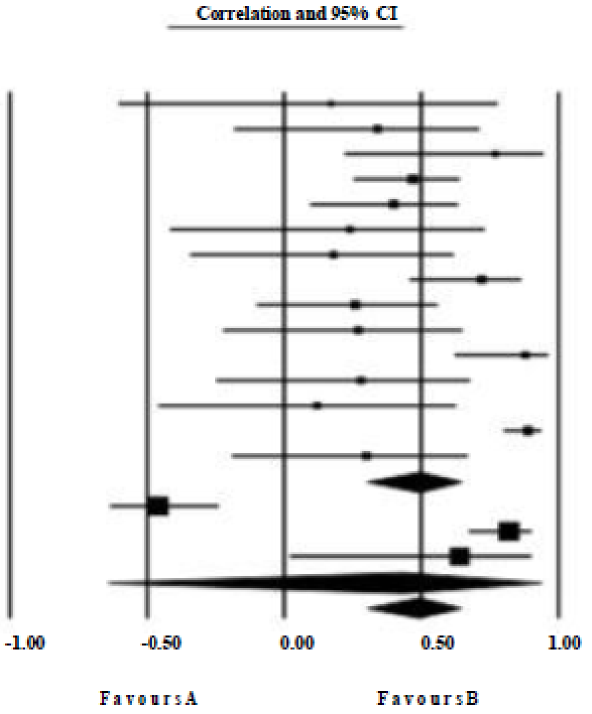

Note: A = Aerobic exercises; E = exercises; H = High intensity interval training; P = psychoeducation; Y = yoga; RCT = randomized controlled trials/controlled trials EX = longitudinal studies with an intervention, no control group; L = observational-longitudinal studies; CS = observational—cross sectional studies.

Table S27

Overall Effects for All Randomized Controlled Trials Included - General Psychopathology

Meta Analysis

Studyname	Std diff in means	Standard error	Variance	Lower limit	Upper limit	Z-Value	p-Value
	Statistics for each study						
Fisher et al., 2020	0.393	0.469	0.220	-0.526	1.312	0.838	0.402
Kaltsatou et al., 2015	0.337	0.362	0.131	-0.372	1.046	0.931	0.352
Romain et al., 2019	2.253	0.317	0.100	1.631	2.874	7.107	0.000
Scheewe et al., 2013	0.706	0.330	0.109	0.059	1.353	2.138	0.033
Shimada et al., 2019	0.615	0.368	0.135	-0.106	1.336	1.673	0.094
Silva et al, 2015 (aerobic)	3.275	0.657	0.432	1.987	4.562	4.984	0.000
Silva et al, 2015 (resistance)	5.095	0.824	0.680	3.480	6.711	6.181	0.000
Svatkova et al., 2015	0.703	0.359	0.129	-0.001	1.406	1.958	0.050
	1.511	0.423	0.179	0.682	2.340	3.573	0.000

Std diff in means and 95% CI

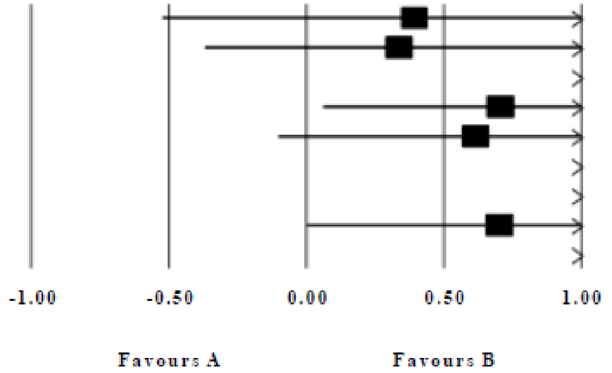

Table S28

Moderating Effects of the Type of Physical Activity Intervention in Randomized Controlled Trials – General Psychopathology

Meta Analysis

Group by Intervention	Study name	Std diff in means	Standard error	Variance	Lower limit	Upper limit	Z-Value	p-Value
Aerobic Exercises	Shimada et al., 2019	0.615	0.368	0.135	-0.106	1.336	1.673	0.094
Aerobic Exercises	Silva et al, 2015 (aerobic)	3.275	0.657	0.432	1.987	4.562	4.984	0.000
Aerobic Exercises		1.889	1.328	1.765	-0.714	4.493	1.422	0.155
Exercises	Fisher et al., 2020	0.393	0.469	0.220	-0.526	1.312	0.838	0.402
Exercises	Kaltsatou et al., 2015	0.337	0.362	0.131	-0.372	1.046	0.931	0.352
Exercises	Scheewe et al., 2013	0.706	0.330	0.109	0.059	1.353	2.138	0.033
Exercises	Silva et al, 2015 (resistance)	5.095	0.824	0.680	3.480	6.711	6.181	0.000
Exercises	Svatkova et al., 2015	0.703	0.359	0.129	-0.001	1.406	1.958	0.050
Exercises		1.218	0.516	0.266	0.206	2.229	2.359	0.018
Overall		1.306	0.481	0.231	0.363	2.248	2.714	0.007

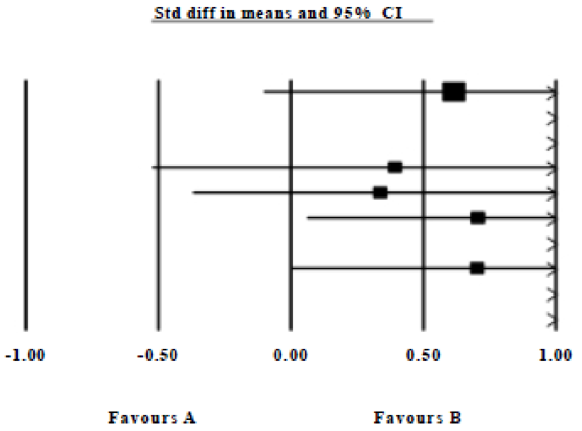

Note: Aerobic Exercises = studies included aerobic exercises interventions; Exercises = studies included interventions with various types of exercise, other than aerobic exercise or HIIT (e.g., resistance exercise).
